# Long COVID: Association of Functional Autoantibodies against G-Protein-Coupled Receptors with an Impaired Retinal Microcirculation

**DOI:** 10.3390/ijms23137209

**Published:** 2022-06-29

**Authors:** Charlotte Szewczykowski, Christian Mardin, Marianna Lucio, Gerd Wallukat, Jakob Hoffmanns, Thora Schröder, Franziska Raith, Lennart Rogge, Felix Heltmann, Michael Moritz, Lorenz Beitlich, Julia Schottenhamml, Martin Herrmann, Thomas Harrer, Marion Ganslmayer, Friedrich E. Kruse, Martin Kräter, Jochen Guck, Robert Lämmer, Matthias Zenkel, Andreas Gießl, Bettina Hohberger

**Affiliations:** 1 Department of Ophthalmology, Universitätsklinikum Erlangen, Friedrich-Alexander-Universität Erlangen-Nürnberg, 91054 Erlangen, Germany; charlotte.sz@yahoo.com (C.S.); christian.mardin@uk-erlangen.de (C.M.); jakob.hoffmanns@extern.uk-erlangen.de (J.H.); thorakatharina.schroeder@extern.uk-erlangen.de (T.S.); franziraith@gmx.de (F.R.); roggelennart@aol.com (L.R.); felix-heltmann@web.de (F.H.); michael.felix.moritz@hotmail.de (M.M.); lorenzbeitlich@gmail.com (L.B.); julia.schottenhamml@fau.de (J.S.); friedrich.kruse@uk-erlangen.de (F.E.K.); robert.laemmer@uk-erlangen.de (R.L.); matthias.zenkel@uk-erlangen.de (M.Z.); andreas.giessl@uk-erlangen.de (A.G.); 2 Research Unit Analytical BioGeoChemistry, Helmholtz Zentrum München-German Research Center for Environmental Health, 85764 Neuherberg, Germany; marianna.lucio@helmholtz-muenchen.de; 3 Berlin Cures GmbH, 10719 Berlin, Germany; gwallukat@berlincures.de; 4 Department of Internal Medicine 3, Universitätsklinikum Erlangen, Friedrich-Alexander-Universität Erlangen-Nürnberg, 91054 Erlangen, Germany; martin.herrmann@uk-erlangen.de (M.H.); thomas.harrer@uk-erlangen.de (T.H.); 5 Deutsches Zentrum für Immuntherapie (DZI), Friedrich-Alexander-Universität Erlangen-Nürnberg (FAU) and Universitätsklinikum Erlangen, 91054 Erlangen, Germany; 6 Department of Internal Medicine 1, Universität of Erlangen-Nürnberg, Friedrich-Alexander-Universität Erlangen-Nürnberg, 91054 Erlangen, Germany; marion.ganslmayer@uk-erlangen.de; 7 Max Planck Institute for the Science of Light & Max-Planck-Zentrum für Physik und Medizin, 91058 Erlangen, Germany; martin.kraeter@mpl.mpg.de (M.K.); jochen.guck@mpl.mpg.de (J.G.)

**Keywords:** functionally GPCR autoantibodies, COVID-19, Long-COVID syndrome, chronic fatigue syndrome, OCT–angiography, glaucoma

## Abstract

Long COVID (LC) describes the clinical phenotype of symptoms after infection with the severe acute respiratory syndrome coronavirus 2 (SARS-CoV-2). Diagnostic and therapeutic options are limited, as the pathomechanism of LC is elusive. As the number of acute SARS-CoV-2 infections was and is large, LC will be a challenge for the healthcare system. Previous studies revealed an impaired blood flow, the formation of microclots, and autoimmune mechanisms as potential factors in this complex interplay. Since functionally active autoantibodies against G-protein-coupled receptors (GPCR-AAbs) were observed in patients after SARS-CoV-2 infection, this study aimed to correlate the appearance of GPCR-AAbs with capillary microcirculation. The seropositivity of GPCR-AAbs was measured by an established cardiomyocyte bioassay in 42 patients with LC and 6 controls. Retinal microcirculation was measured by OCT–angiography and quantified as macula and peripapillary vessel density (VD) by the Erlangen-Angio Tool. A statistical analysis yielded impaired VD in patients with LC compared to the controls, which was accentuated in female persons. A significant decrease in macula and peripapillary VD for AAbs targeting adrenergic β2-receptor, MAS-receptor angiotensin-II-type-1 receptor, and adrenergic α1-receptor were observed. The present study might suggest that a seropositivity of GPCR-AAbs can be linked to an impaired retinal capillary microcirculation, potentially mirroring the systemic microcirculation with consecutive clinical symptoms.

## 1. Introduction

The severe acute respiratory syndrome coronavirus 2 (SARS-CoV-2), observed in Wuhan (2019) [1], reached a worldwide pandemic level by March 2020 [2]. The virus, which spread to over 418 million cases with over 5.8 million deaths (numbers from the World Health Organization (WHO), accessed 18 February 2022) [3], has caused a number of pneumonia cases [1]. This global health problem requires intensive research on its pathogenesis, clinical features (phenotyping) and therapeutic approaches. Following acute coronavirus disease 2019 (COVID-19), patients are at risk of suffering from Long COVID (LC). LC can affect each gender and age independently of the severity of the acute COVID-19 disease [4,5,6,7]. LC covers a wide range of symptoms, e.g., fatigue, post-exertional malaise (PEM), loss of concentration, myalgias, cognitive impairment (“brain fog”), shortness of breath, neurological or cardiovascular symptoms (e.g., myocardial inflammation, palpitations and tachycardia potentially presenting as postural orthostatic tachycardia syndrome (POTS)) [4,6,8,9]. LC does not only restrict patients’ quality of life, but it also has a great impact on general healthcare and the economy considering its incidence [5]. Depending on the study design, cohort incidences of 2.3% to over 91% were reported in the literature [8,10]. However, numerous studies suggest that more than half of patients with acute COVID-19 infection could be affected by LC [5,6,7].

The pathogenesis of LC is still under investigation, yet several factors and molecular pathways have been identified at this time point. It is assumed that LC has an involvement of autoimmunity [11,12,13]. The virus itself, its contact to the endothelium with consecutive endotheliitis and endothelial dysfunction [14,15,16], and the involvement of humoral response and autoantibodies seem to interplay in LC. It is hypothesized that systemic blood circulation is impaired [17], potentially with the participation of microclots [14,18] and neutrophils [19,20]. In addition, the direct and indirect influence of the virus by attacking the blood vessels should be considered. Post-mortem biopsies of the endothelium showed not only components of SARS-CoV-2 inside the cells but also several indications of endothelial involvement with the virus, such as endotheliitis and cell necrosis [16].

Visualizing the impaired microcirculation would have a great impact on daily clinical operations by bringing these molecular research data from the bench to the bedside. Optical Coherence Tomography–Angiography (OCT–A) [21,22] is an innovative technique, enabling the non-invasive visualization and quantification of the regional capillary system. OCT–A is an extended version of OCT. OCT–A detects moving objects in the scanning area (red blood cells in the vessels; RBCs) and visualizes this information via a binary code (white moving elements; black not-moving elements). This time-dependent OCT signal can be quantified by the parameter of vessel density (VD). VD represents the ratio of white to black signals. The lower the VD, the less RBC movement that can be observed. Thus, a lower VD can be seen as restricted or missing blood flow or can be a sign of capillary death. This OCT–A analysis can be performed in a detailed way; three microvascular layers can be differentiated within the whole retina: the superficial vascular plexus (SVP), the intermediate capillary plexus (ICP), and the deep capillary plexus (DCP). After COVID-19 infection, a reduced retinal VD was observed [17,23]. Histopathological correlates might be seen in the presence of ghost vessels in the post-mortem eyes of patients with COVID-19 [24]. We assume that the restriction of retinal microcirculation might be representative of the systemic microcirculation, which can be easily and non-invasively monitored by ophthalmologists.

In a previous case report, the impaired retinal microcirculation was improved after the neutralization of functionally active autoantibodies against G-protein-coupled receptors (GPCR-AAbs) [12]. These GPCR-AAbs were observed in patients after COVID-19 disease, targeting the adrenergic β2-receptor (β2-AAb), the angiotensin-II-type-1 receptor (AT1-AAb), the MAS-receptor (MAS-AAb), the muscarinergic M2 receptor (M2-AAb), the Nociceptin-receptor (Noci-AAb), the endothelin-A receptor (ETA-AAb), and the α1-adrenergic receptor (α1-AAb) [13]. Going along with these molecular and microvascular improvements, the LC symptoms of the patient were resolved [12]. The basis of this experimental therapy was the research data on a link of the β2-AAb to retinal microcirculation in glaucoma patients [25]. Thus, it was assumed that a seropositivity of GPCR-AAbs might be linked to LC symptoms. GPCR-AAbs are associated with various diseases such as chronic fatigue syndrome [26], dilatative cardiomyopathy [27], preeclampsia [28] and, recently, glaucoma [25]. We hypothesize that not only might a seropositivity of β2-AAb be linked to an impaired retinal microcirculation and thus LC symptoms, but also a seropositivity of other functionally active GPCR-AAbs might interplay in the context of a restricted retinal and systemic microcirculation. The aim of this study was to correlate a seropositivity of functionally active GPCR-AAbs with microcirculation, mapped by OCT–A in patients with LC.

## 2. Results

All patients with LC that were included in the study showed a seropositivity for GPCR-AAb, but none of the control subjects did. The results from the statistical models were provided as beta estimates and for the different effects of the least-squares means (LS means) for overall and sectorial VD. We revealed remarkable differences in all the VDs when we compared the LC patients to the controls. Overall, the LS mean VDs were 29.55 ± 0.1 (SVP), 21.27 ± 0.1 (ICP), 23.03 ± 0.1 (DCP), and 40.06 ± 0.3 (peripapillary region) in patients with LC. For the controls, the LS mean VDs were 30.63 ± 0.4 (SVP), 22.41 ± 0.3 (ICP), 24.54 ± 0.4 (DCP), and 42.39 ± 1.0 (peripapillary region). A significant reduction in VD was observed in the SVP (*p*-value = 0.0061), ICP (*p*-value = 0.0017), DCP (*p*-value = 0.0005), and peripapillary region (*p*-value = 0.0286) of patients with LC compared to the controls (Table 1).

**Figure 1:**
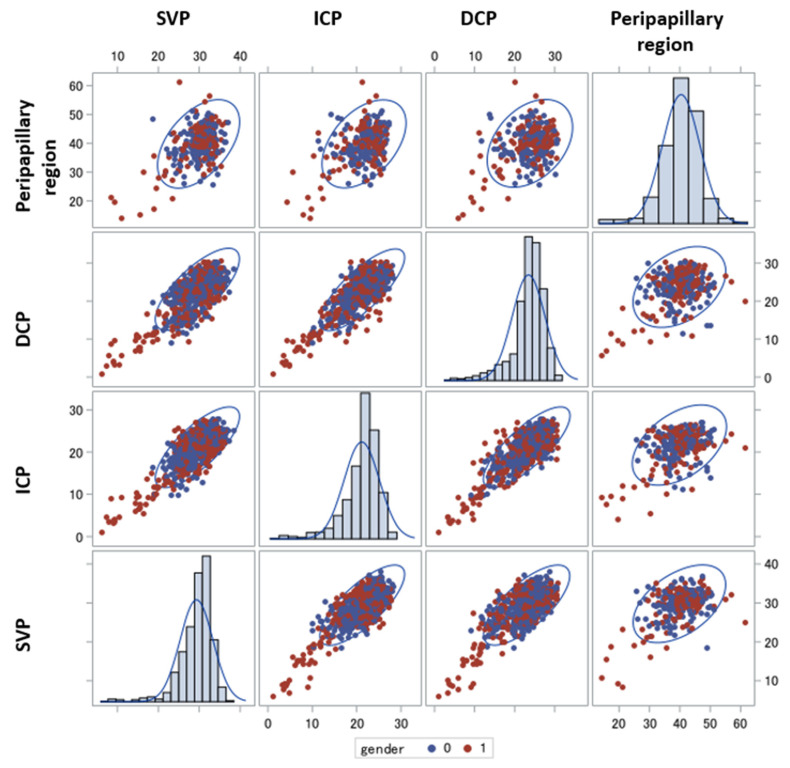
Vessel density differences in patients with Long COVID regarding gender. Relations between vessel density (SVP, ICP, DCP and peripapillary region) of patients with Long COVID (LC) colored by gender (male, blue; female, red), with 95% prediction ellipse; female patients with LC showed lower VD data; SVP—superficial vascular layer; ICP—intermediate capillary plexus; DCP—deep capillary plexus.

In Figure 1, the distribution of the VD of each retinal layer considering gender in patients with LC is presented. We denoted that especially women have low levels for each of the comparison pairs of GPCR-AAbs. In particular, there was a significant decrease in VD in the SVP and ICP for the female patients in comparison with the males (*p* = 0.017 [CI: 0.11; 1.11], and *p* = 0.0003 [CI: 0.42; 1.11], respectively). A similar tendency was observed in all the other layers.

The frequency distributions (with the percentages and cumulative values) of the GPCR-AAbs (β2-AAb, AT1-AAb, MAS-AAb, M2-AAb, Noci-AAb, α1-AAb, ETA-AAb) in patients with LC are presented in Table 2. The seropositivities of β2-AAb, M2-AAb, AT1-AAb, and MAS-AAb were the predominantly observed ones.

**Figure 2:**
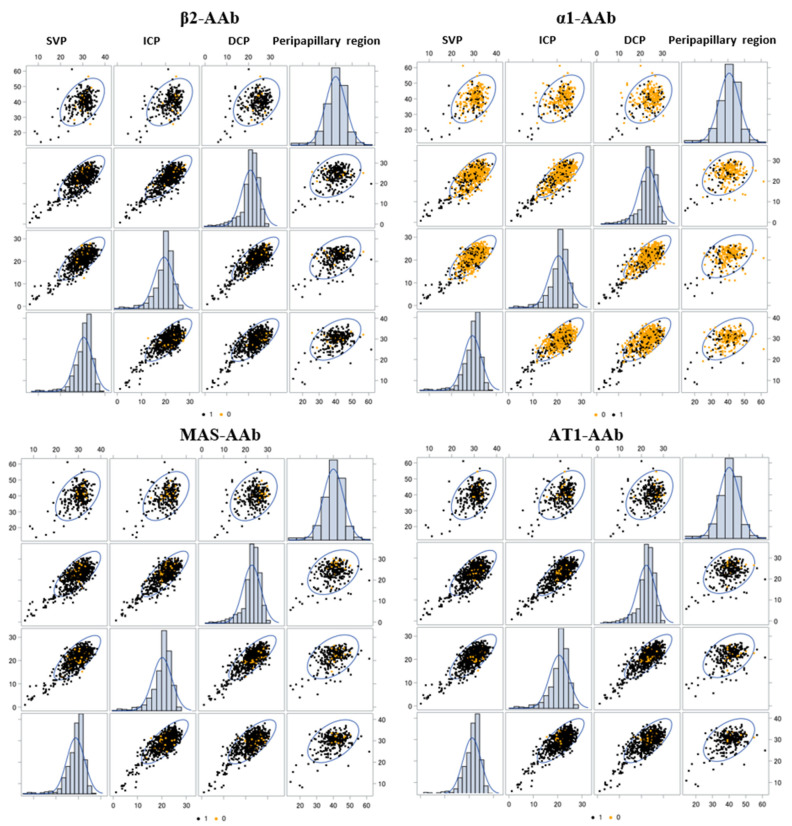
Vessel density in relation to seropositivity and seronegativity of specific autoimmune antibodies. Association between the vessel densities of the SVP, ICP, DCP, and peripapillary region of patients with Long COVID considering a seropositivity of β2-AAb, α1-AAb and MAS-AAb with the 95% prediction ellipse. Black = Seropositivity (1), Yellow = Seronegativity (0); SVP—superficial vascular layer; ICP—intermediate capillary plexus; DCP—deep capillary plexus.

The overall LS mean VD results of the different mixed models are presented in Table 3 for the SVP, ICP, DCP, and peripapillary region in patients with LC considering a seropositivity for each GPCR-AAb, respectively (Table 3). Figure 2 represents scatter plots of VD of each retinal layer as a function of a seropositivity of β2-AAb, α1-AAb, AT1-AAb, and MAS-AAb in patients with LC. We denoted a linear positive association between all of them, with a remarkable predominance of the seropositivity of the GPCR-AAbs (except for α1-AAb).

The models with age and gender as covariates showed substantial differences in the LS mean VD for a seropositivity of α1-AAb. A significant different VD of SVP ([CI: 0.86; 4.22], *p* = 0.0035), of DCP ([CI: 0.47; 43.98], *p* = 0.0138), and of the peripapillary region ([CI: 1.09; 6.34], *p* = 0.0062) were observed in patients with LC considering a seropositivity of α1-AAb, respectively. In addition, significant differences in VD of the DCP were yielded when comparing patients with LC and a seropositivity with the seronegativity of MAS-AAb (CI: 2.29; 6.63, *p* = 0.0001). The LS mean VD of the ICP was significantly impaired in patients with LC regarding a seropositivity of β2-AAb (CI: 0.08; 2.01, *p* = 0.0344). A significant different LS mean VD of DCP was observed in patients with LC considering a seropositivity of AT1-AAb (CI: 0.45; 5.09; *p* = 0.0197). No significant differences in the macula and peripapillary LS mean VD were observed for patients with a seropositivity of the remaining GPCR-AAbs. No prominent differences were observed for Noci-AAb, M2-AAb or ETA-AAb in the VD data of the SVP, ICP, DCP, and peripapillary region (*p* > 0.05).

**Figure 3:**
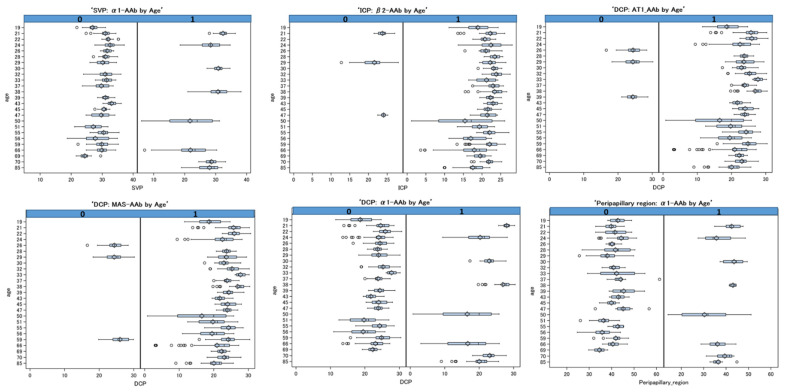
Box plots of vessel density of SVP, ICP, DCP and peripapillary region with the variable age for the mixed model with repetition. The two panels show the distribution of vessel density for patients with Long COVID and a seropositivity (=1) and seronegativity (=0) for α1-AAb, AT1-AAb, MAS-AAb, and β2-AAb. The effects are significant (*p* < 0.05); SVP—superficial vascular layer; ICP—intermediate capillary plexus; DCP—deep capillary plexus.

In Figure 3, the age’s trend is represented for the VD of the SVP, DCP, ICP and peripapillary region, respectively. It is divided into two panels for the seropositivity and seronegativity for each significant GPCR-AAb. The different box plots underline the different trends among the covariate age, taking into account all the repetitions. Seropositivities for MAS-AAb, β2-AAb, AT1-AAb, and α1-AAb were observed in all analyzed patients’ age groups (19–85 years) with a significant negative trend (*p* < 0.05). All the age variable estimations are presented in Supplementary Table S1.

**Figure 4:**
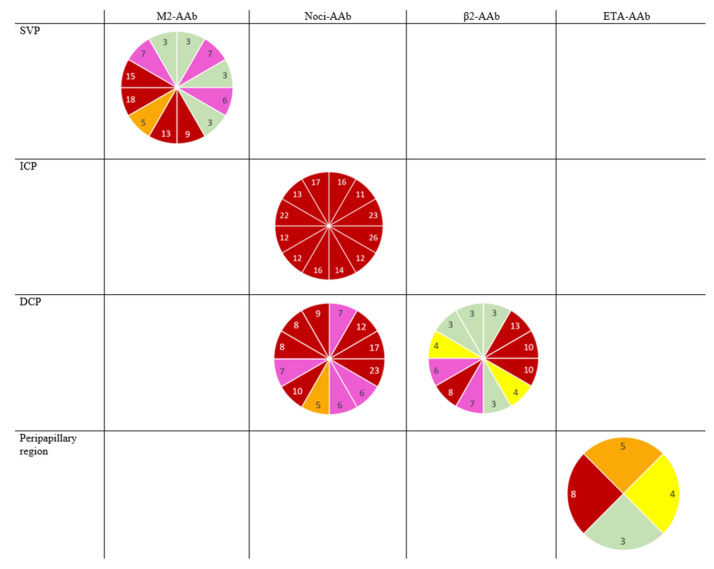
Schematic color-coded sketch of the number of significant interactions: sectorial vessel density of SVP, ICP, DCP (*n* = 12), and peripapillary region (*n* = 4) is presented considering the effect of the M2-AAb, Noci-AAb, β2-AAb, and ETA-AAb; red, *n* ≥ 8; pink, *n* = 6–7; orange, *n* = 5; yellow, *n* = 4; green, *n* = 2–3; gray, *n* = 0–1; SVP—superficial vascular plexus of the macula; ICP—intermediate capillary plexus of the macula; DCP—deep capillary plexus of the macula.

When introducing the interaction terms (sector with GPCR-AAb) into the models, we observed significant differences between different sectorial VDs in the ICP and the DCP (Noci-AAb), in the SVP (M2-AAb), in the peripapillary region (ETA-AAb), and in the DCP (β2-AAb). All the *p*-values (adjusted with Tukey–Kramer) for the multiple comparisons are presented in Supplementary Table S2. The qualitative analysis of the number of significant interactions between the sectorial macula and peripapillary VDs in all the retinal layers are shown color-coded in Figure 4 (red, *n* ≥ 8; pink, *n* = 6–7; orange, *n* = 5; yellow, *n* = 4; green, *n* = 2–3; gray, *n* = 0–1) for patients with LC, considering a seropositivity of α1-AAb and β2-AAb.

When the models were additionally corrected with the FAZ variables, the results confirmed the significance of the LS mean differences of α1-AAb. Moreover, we observed additional significant effects of β2-AAb, Noci-AAb and M2-AAb on the VD of different retinal layers (Supplementary Table S3a). The estimates of the VD of each retinal layer with the variable FAZ of the SVP, ICP, and DCP as covariates (including age and gender as further covariates) are presented in Supplementary Table S3b. Remarkably, a seropositivity of β2-AAb showed significant effects on the VD of the ICP with the covariates FAZ of the SVP (*p* = 0.0025), FAZ of the ICP (*p* = 0.0037), and FAZ of the DCP (*p* = 0.0354), respectively. In addition, a seropositivity of M2-AAb showed a remarkable effect on the VD of the ICP with the covariates FAZ of the SVP (*p* = 0.0011), FAZ of the ICP (*p* = 0.001), and FAZ of the DCP (*p* = 0.0389), respectively.

## 3. Discussion

Due to the COVID-19 pandemic, the acute SARS-CoV2 infection and LC are a challenge for the healthcare system. According to the WHO definition, LC is characterized as ongoing or new clinical symptoms four weeks after a SARS-CoV-2 infection. This clinical feature summarizes the term ‘ongoing COVID’ (week 4–12 after SARS-CoV-2 infection) and ‘Post-COVID-19 syndrome’ (>week 12 after SARS-CoV-2 infection) [29]. A part of the LC symptoms might be triggered by autoimmune pathways [11,12,13]. A hyperactive immune response, endothelial affections and an impaired microcirculation (e.g., due to NETs, platelet activation, microclots) interplay in this pathogenesis [17,18,30]. Viral affection of the endothelium [16] and humoral immune response might be the basis for AAb formation. As GPRC-AAb targeting vasoactive receptors or proteins [31] were observed in the sera of patients after COVID-19 infection, and one of them (β2-AAb) is known to impair retinal microcirculation in glaucoma patients, a link of GPCR-AAb with microcirculation was assumed. This hypothesis is supported by the clinical finding of a successful experimental therapy of neutralization of GPCR-AAb in glaucoma patients with LC [12]. Going along with the improvement of microcirculation (mapped in the retina using OCT–A), LC symptoms were ameliorated in this patient. The ophthalmologic method (OCT–A) enables the visualization of regional microcirculation (retina), potentially being a mirror of the systemic capillary system. As it is known that a seropositivity of β2-AAb might have a harmful impact on microcirculation in glaucoma disease, it was the aim of this study to investigate a potential further impact of GPCR-AAb on microcirculation in patients with LC, being mapped in the retina using OCT–A. The present data showed that all of the included patients with LC showed a varying composition of each GPCR-AAb. In terms of microcirculation, female patients with LC in particular showed more restricted retinal microvasculature than male subjects. Significantly different VDs were observed for patients with LC and a seropositivity of α1-AAb, AT1-AAb, MAS-AAb, and β2-AAb in different retinal layers compared to seronegative ones. Thus, we assume an impact of these GPCR-AAb on microcirculation in LC.

Several autoantibodies were observed during or after COVID-19 infection [11,12,13]. Yet, especially functionally active AAbs might have a harmful effect on the human body. They might disturb physiological cellular pathways and intercellular communications, potentially in the presence of further cofactors. SARS-CoV-2 invades via the binding of its spike protein to the human angiotensin-converting enzyme 2 (ACE-2) on human cells [32]. ACE-2 is a protein that is present in a wide range of variation in human tissues, including the heart, lung and blood vessels [33]. It is notable that the virus appears to be able to gain entry into various organs, particularly via epithelial and endothelial cells [34], thus capillary microcirculation is already attacked during acute infection. Two subtypes of ACE have been described up to now: ACE-1 induces the conversion of angiotensin I to its vasoactive form (angiotensin II) by separating the carboxy terminal dipeptide of angiotensin I. ACE-2 is a monocarboxypeptidase, converting angiotensin II to the peptide angiotension-(1–7). The latter binds to the MAS-related GPCR (MRGPR), influencing the effect of angiotensin II on the AT1 receptor subtype, and thus on the downregulation of the renin–angiotensin–aldosterone system (RAS), accompanied by anti-inflammatory and anti-fibrotic effects [35]. The MRGPR is a large family of GPCRs that is mainly expressed in immune cells such as mast cells and primary sensory neurons [36,37,38]. MRGPR dysregulation seems to be involved in itches [36], nociception [38], mast-cell degranulation [39], sleep [40] and microcirculation [41]. The binding of SARS-Cov-2 to the ACE-2 enzyme might mimic an ACE-2-deficiency with the consequent risk of thrombosis and the inflammation process [42] as ACE-2 (via degradation of angiotensin II) and Ang-(1–7) act anti-inflammatorily on endothelial cells [35,43]. Even angiotensin II can act as an inflammatory cytokine itself [44]. In addition, fibrosis might occur [34]. The assumption that these viral-induced pathologies and symptoms are strongly dependent on ACE-2 is underlined by the increased incidence of severe and lethal infections in patients with pathologies of the RAS (e.g., diabetics, hypertension) [42].

The association of GPCRs and the RAS and SARS-CoV-2 and the RAS via ACE-2, respectively, might be a factor in LC pathogenesis. The RAS systems act via the binding and activation of angiotensin II to the AT1-receptor and angiotensin-(1–7) to the MAS-receptor [35]. Functionally active autoantibodies were observed in patients with LC, targeting and activating these GPCRs. A seropositivity of GPCR-AAb was already described in several systemic and local disorders earlier (Table 4). AT1-AAb and MAS-AAb were even known from other diseases, going along with common features: AT1-AAbs have already been linked to cardiovascular diseases, including hypertension and preeclampsia [28,45]. In vitro data showed an impact of α1-AAb and AT1-AAb on mast-cell modulation [46], being a clinical feature in patients with LC (mast-cell activation) [47]. MAS-AAbs were seen in a patient after tumor chemotherapy [48]. Further muscarinic-AAbs (M1-AAb, M2-AAb, M3-AAb, M3-AAb) and β2-AAbs were already observed in the sera of patients with chronic fatigue (ME/CFS) [26,49]. A seropositivity of AAb against adrenergic (α1, β2), muscarinic (especially M2 and M4) and nociceptin receptors was described in patients with POTS [50,51].

The present study yielded a varying composition of GPCR-AAbs in the participants with LC. The seropositivities of α1-AAb, AT1-AAb, MAS-AAb, and β2-AAb were linked to an altered microcirculation, mapped in the retina using OCT–A. This data goes along with recent data of an impaired retinal microcirculation in glaucoma patients with a β2-AAb seropositivity [25]. As retinal microcirculation might be seen as a mirror to the human body, the use of OCT–A offers great options in the diagnosis and monitoring of ophthalmic and systemic disorders. It is well known that OCT–A data can be useful in interdisciplinary patient care [21,22]. This ophthalmic method is a novel and non-invasive method, which can visualize and quantify retinal microcirculation [52]. The physical basis of OCT–A is the scattering of moving RBCs in capillaries [53,54]. The scattering of each RBC is summarized by each refractive index, its shape and size [55]. Thus, impaired VD data can be interpreted as very low RBC flow or occluded capillary vessels (ghost vessels). Previous studies showed an impaired retinal microcirculation that was dependent on the severity of the acute COVID-19 disease [17,23]. The greatest impairment of VD was observed in the intermediate layer, where ACE-2 receptors have already been observed at the molecular level [56]. To the best of our knowledge, this is the first study investigating retinal microcirculation in patients with LC considering the impact of a seropositivity of functionally active GPCR-AAbs. There is evidence that reduction, elimination or neutralization of the GPCR-AAbs goes along with the improvement of clinical features in patients with a seropositivity of those GPCR-AAbs. Either therapeutic plasma exchange (TPE) [57], immunoadsorption [25,58], immunoglobulin therapy (IVIg) [59] or the aptamer BC 007 [12] were observed to improve the patients’ symptoms in each clinical entity. It can be assumed that GPCR-AAbs have a harmful impact on diseases with autoimmune impacts (e.g., POTS, ME/CFS, Long COVID).

**Figure 5:**
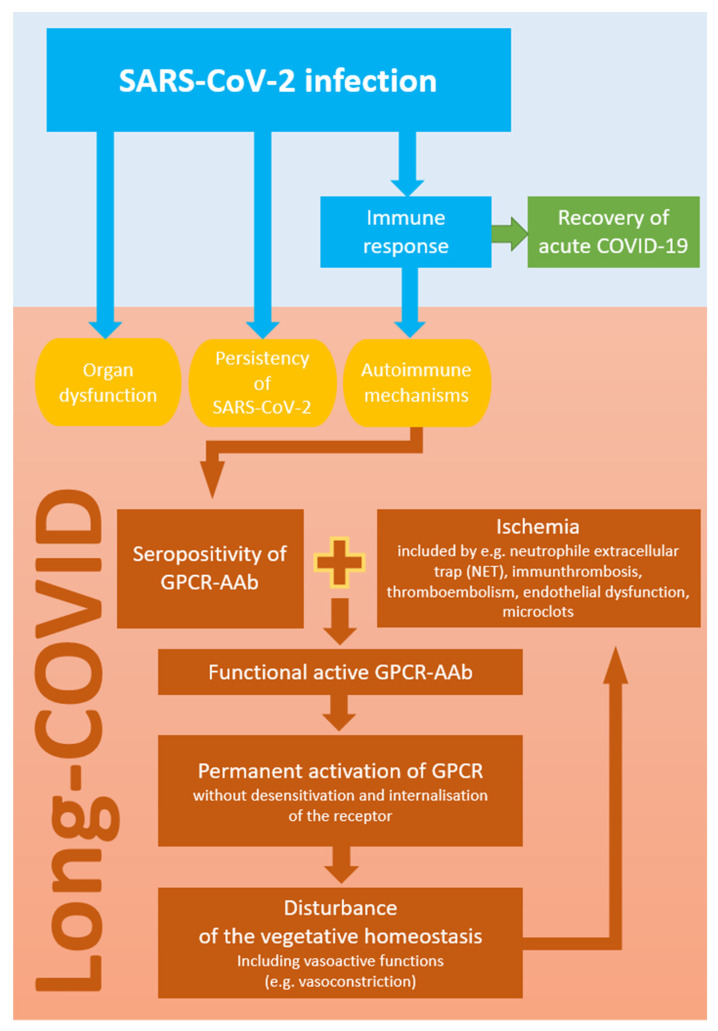
Schematic hypothesis of GPCR-AAb-mediated autoimmunity in Long COVID: a seropositivity of GCPR-AAb and ischemia might induce a vicious circulus.

Up to now, the exact pathogenesis of the generation of these GPCR-AAbs is still elusive. Only in Chagas’ cardiomyopathy is a molecular mimicry after an infection with Trypanosoma cruzi known [60]. It might be reasonable that, via homologies of the pathogen and physiological human tissue, the production of GPCR-AAbs might arise. Further viral reproduction itself could insert molecules of the endothelial cell surface into the virion, being recognized by the humoral response. We assume that additional conditions or cofactors are necessary for the functional activity of GPCR-AAbs [61]. The molecular consequences of ligand–receptor interactions are determined by both the ligand (GPCR-AAb) and the receptor itself, which can be modified by pre-existing cofactors (e.g., by ischemia via change of the receptor conformation) [62]. Previous in vitro data showed that agonistic AT1-AAb can induce vascular contraction only in the presence of ischemia or inflammation [63]. The presence of GCPR-AAb alone was not observed to act as a harmful agent. The authors stated that “ischemia represents a key permissive factor for their (i.e., GPCR-AAb) vasoconstrictive actions”. These effects were also seen for β2-AAb. In rocker cultures (with good oxygenation due to a slow movement of the cells), β2-AAb or hydrophilic β-adrenergic agonists show only marginal functional activity. Contrarily, if the cells were cultured under stationary conditions (low oxygenation), then functional activity of the β2-AAb and hydrophilic β-adrenoceptor agonists were observed [64]. After the binding of the GPCR-AAb, the receptor and its signal cascade are permanently activated, as GCPR-AAb activation shows no desensitization or internalization of the GPCR contrary to physiological ligands. Consequently, the cellular homeostasis of Ca^2+^ is disturbed and apoptosis can be initiated [65,66,67]. In summary, ischemia-induced GCPR-AAb activity might show its harmful pathogenic impact via the lack of tachyphylaxia [68,69,70] (Figure 5).

The present study is not without limitations. The study size is small, yet even in this cohort we revealed significant effects on VD by considering gender and a seropositivity of GPCR-AAb. In addition, the selection of patients with LC is a challenging topic as the actual diagnostic procedure is based on clinical symptoms of LC and the exclusion of other diseases. Thus, it would be great to have in vivo or in vitro biomarkers.

**Table 4:** Seropositivity of GPCR-AAbs in local and systemic disorders.

	α1-AAb	β1-AAb	β2-AAb	M2-AAb	AT1-AAb	ETA-AAb	Noci-AAb	MAS-AAb	Reference
Alzheimer’s disease	x		x						[71]
Vascular dementia	x		x			x			[71]
Chagas’ disease		x	x	x					[27,72]
DCM		x		x					[27]
Preeclampsia					x				[28]
Hypertension	x				x				[45]
Myocarditis		x							[68]
Glaucoma			x						[25]
Post COVID	x		x	x	x	x	x	x	[13]
CFS			x						[26]
POTS	x	x	x	x			x		[50,51]
TAO	x				x	x			[73]
Type 2 diabetes	x	x			x				[74]
Allergic Asthma			x						[75]
After Chemotherapy	x			x				x	[48]

DCM = dilated cardiomyopathy; TAO = thromboangitis obliterans; POTS = postural orthostatic tachycardia syndrome; CFS = chronic fatigue syndrome; α1-AAb = autoantibody targeting α1-adrenergic receptor; β1-AAb = autoantibody targeting β1-adrenergic receptor; β2-AAb = autoantibody targeting β2-adrenergic receptor; M2-AAb = autoantibody targeting muscarinic-M2 receptor; AT1-AAb = autoantibody targeting angiotensin-II-AT1 receptor; ETA-AAb = autoantibody targeting endothelin-A receptor; Noci-AAb = autoantibody targeting nociceptin-like opioid receptor.

## 4. Material and Methods

### 4.1. Participants

A prospective study with 92 eyes of 48 persons was performed at the Department of Ophthalmology, Friedrich-Alexander-Universität Erlangen-Nürnberg (FAU): 83 eyes of 42 patients after COVID-19 infection (female: *n* = 20, age 41.8 ± 16; male: *n* = 22, age 41.91 ± 19) and 9 eyes of 6 controls (female: *n* = 2, age of 38.5 ± 19; male: *n* = 4, age 39.5 ± 18) were included. A SARS-CoV-2 infection was confirmed by a real-time, reverse-transcription–polymerase-chain-reaction (PCR) test. LC was defined as ongoing and/or new clinical symptoms after confirmed SARS-CoV-2 infection. The most frequent symptoms were brain fog (76%), fatigue (64%), and cardiovascular abnormalities (e.g., tachycardia, bradycardia, hypertension; 61%). As LC is assumed to summarize several subtypes, only patients with clinical symptoms of LC, a positive PCR test for SARS-Cov-2 infection, and a seropositivity of GPCR-AAb were included in the present study. The mean time after confirmed SARS-CoV-2 infection was 200 ± 110 days (range 34–484 days). No eyes showed any local or systemic disorders with retinal affection. Furthermore, the best-cured visual acuity (BCVA) and intraocular pressure (IOP) were measured. Axial length was measured by IOL Master (Zeiss, Oberkochen, Germany). The study has been approved by the local ethics committee and performed in accordance with the tenets of the Declaration of Helsinki. All patients signed a written informed consent form.

### 4.2. Measurement of GPCR-AAb by a Cardiomyocyte Bioassay

An established cardiomyocyte bioassay was used to analyze the presence of GPCR-AAbs. This method was established by Wallukat and Wollenberger [75], and is described in detail in [76,77]. Briefly summarized, the cardiomyocytes from newborn Wistar rats were obtained and transferred to a cardiomyocyte cell culture. To obtain the GPCR-AAbs containing IGg, the patients’ serum was dialyzed against a dialyzing buffer of 0.15 M NaCl, 10 mM phosphate buffer at a pH of 7.4 (Membra-Cel MD 44, 14 kDa, Serva), stored at −20° until further utilization. The dialysate (40 µL) was added to the bioassay and incubated for 60 min. Changes in basal frequency of the rat myocytes (cut-off 1.8 beats/15 s), expressing the target structures (GPCR) were used to identify GPCR-AAbs: Either an increase in the beating rate (positive chronotropic) or a decrease in the beating rate (negative chronotropic) was measured in the presence of GPCR-AAbs. A positive chronotropic effect could be observed for β2-AAb, α1-AAb, AT1-AAb, and Noci-AAb. A negative chronotropic effect was described for AAbs targeting the M2-AAb, MAS-AAb, and ETA-AAb. For specification of the GPCR-AAb subtype, specific blockers were added to the bioassay: 0.1 μM of ICI118,551 (β2-AAb), 1µM of atropin (M2-AAb), 1µM losartan (AT1-AAb), 1 μM of A779 (MAS-AAb), 0. 1 μM BQ123 (ETA-AAb), 0.1 μM J113397 (Noci-AAb), and 1 μM urapidil or prazosin (α1-AAb). The receptor blockers reversed the effect of the GPCR-AAbs, as shown by either increasing or decreasing (depending on the effect of the GPCR-AAb) chronotropy.

### 4.3. OCT–Angiography (OCT–A)

En face OCT–A (Heidelberg Spectralis II, Heidelberg, Germany) can measure the VD of the macula and the peripapillary region (angle of 15°; lateral resolution of 5.7 µm/pixel). OCT–A scans (2.9 mm × 2.9 mm) correspond to a size of 8.41 mm² of the human retina. In addition, macula microvasculature can be subdivided into three adjacent layers: the superficial vascular plexus (SVP), intermediate capillary plexus (ICP), and deep capillary plexus (DCP). Peripapillary en face OCT–A scans were projected between the retinal nerve fiber layer and inner plexiform layer.

**Figure 6:**
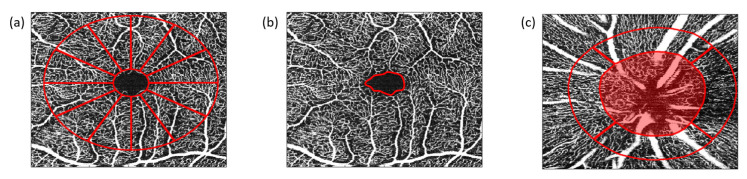
Optical coherence tomography angiography (OCT–A): (**a**) Schematic sketch of quantitative OCT–A analyses of the macula (12 sectors, at 30°), (**b**) foveal avascular zone (FAZ, red), and (**c**) the peripapillary region (4 sectors, at 90°), quantified by the Erlangen-Angio Tool; quantification of vessel density was performed by implementation of the Anatomic Positioning System (APS, part of Glaucoma Module Premium Edition [GMPE], Heidelberg Engineering, Heidelberg, Germany) considering each individual anatomy. The APS can be done by fixation of two points, the fovea and the center of the Bruch’s Membrane Opening (BMO), and the subsequent adjustment of the image to each individual Fovea-to-Bruch’s Membrane Opening-Center (FoBMOC). Peripapillary OCT–A scans were analyzed as BMO-based.

Furthermore, the areas of the foveal avascular zone (FAZ) of the SVP, ICP, and DCP were calculated (Figure 6). After exporting the data by the SP-X1902 software (prototype software, Heidelberg Engineering, Heidelberg, Germany), OCT–A scans were subsequently analyzed using Erlangen-Angio-Tool (EA-tool), coded in MATLAB (The MathWorks, Inc., Natick, MA, USA, R2017b). The EA-tool shows high reliability and reproducibility [52]. The overall and sectorial VD (12 sectors (macula) and 4 sectors (peripapillary)) can be calculated. For an enhanced interpretation of all individual data sets, we implemented the Anatomic Positioning System (APS, part of Glaucoma Module Premium Edition [GMPE], Heidelberg Engineering, Heidelberg, Germany) into the EA-tool. All OCT–A scans were adapted to each individual anatomy. This was performed by the fixation of two points, the fovea and the center of the Bruch’s Membrane Opening, and the subsequent adjustment of the image to each individual Fovea-to-Bruch’s Membrane Opening-Center (FoBMOC) [17,52,78].

### 4.4. Statistics

The data were analyzed by the mixed model (SAS version 9.4, Institute Inc., Cary, NC, USA) taking into consideration the repetitions of the eyes for each sector of the macula OCT–A scans. Three different structures of covariance (unstructured, compound symmetry and ante-dependence) were compared, and the lowest Akaike’s information criterion (AIC) and Bayesian information criterion (BIC) values were chosen. In the first models, only gender and age were set as covariates; later, FAZ variables also were chosen. Additionally, we modeled the interactions of the effects of the sectors by the scans and by FAZ variables. We estimated the least-squares means (LS means) that corresponded to the specified effects for the linear predictor part of the model and the relative confidence limits. The LS means are closer to reality and represent even more real data, when cofactors occur, compared to means. The *p*–values (the α value is set at 0.05) are presented with the respective confidence interval limit (CL). All the CLs and the *p*-values for the multiple comparisons are adjusted with Tukey–Kramer.

## 5. Conclusions

Long COVID is a disease characterized by a variety of clinical symptoms including neurological and vascular ones. As an autoimmune component is assumed to be involved in the pathogenesis of LC, the data of the present study yielded that a seropositivity of GPCR-AAb showed an impact on impaired microcirculation, mapped in the retina using OCT–A in patients with Long COVID. Further research is necessary to elucidate the molecular mechanisms behind this clinical finding.

## Figures and Tables

**Table 1:** Retinal microcirculation of patients with Long COVID (LC) compared to controls, quantified by macula (SVP, ICP, DCP) and peripapillary vessel density of OCT–A scans; SVP—superficial vascular plexus; ICP—intermediate capillary plexus; DCP—deep capillary plexus; SE—standard error. The estimates are represented as the differences between the seronegativity estimate and the seropositivity estimate with the lower and upper confidence limits (CL).

Differences of Least-Squares Means									
	Effect	Group		Estimate	SE	t Value	Pr > |t|	Lower CL	Upper CL
SVP	group	LC	control	−1.09	0.39	−2.81	0.0061	−1.85	−0.32
ICP	group	LC	control	−1.14	0.35	−3.23	0.0017	−1.84	−0.44
DCP	group	LC	control	−1.51	0.42	−3.61	0.0005	−2.35	−0.68
Peripapillary region	group	LC	control	−2.33	1.05	−2.23	0.0286	−4.41	−0.25

**Table 2:** Frequencies and percentages, with their cumulative values for each GPCR-AAb variable: Noci-AAb, β2-AAb, AT1-AAb, α1-AAb, MAS-AAb, M2-AAb, and ETA-AAb. The values are distinguished by seropositivity; absolute frequency represents the actual number of observations in the given interval; percentage frequency represents the result of dividing the absolute frequency of each return interval by the total number of observations. Cumulative frequency and cumulative relative frequency are the results of cumulating the absolute and relative frequencies as we move from the first to the last interval.

GPCR-AAb	Seropositivity (1) Versus Negativity (0)	Frequency	%	Cumulative Freq	Cumulative%
β2-AAb	0	5	6.02	5	6.02
	1	78	93.98	83	100
M2-AAb	0	5	6.02	5	6.02
	1	78	93.98	83	100
AT1-AAb	0	6	7.23	6	7.23
	1	77	92.77	83	100
MAS-AAb	0	6	7.23	6	7.23
	1	77	92.77	83	100
Noci-AAb	0	57	68.67	57	68.67
	1	26	31.33	83	100
α1-AAb	0	63	75.9	63	75.9
	1	20	24.1	83	100
ETA-AAb	0	79	95.18	79	95.18
	1	4	4.82	83	100

**Table 3:** Differences of the LS means (seronegativity versus seropositivity) from the mixed models for overall macula and peripapillary region vessel density considering the GPCR-AAb status. The differences were calculated between the LS mean estimations of seronegativity and seropositivity of GPCR-AAb; moreover, we calculated the lower and upper confidence limits (CLs); SVP—superficial vascular plexus; ICP—intermediate capillary plexus; DCP—deep capillary plexus; SE—standard error; the estimates are represented as the differences between the seronegativity estimate and the seropositivity estimate.

Differences of Least-Squares Means of Vessel Density							
		Estimate	SE	t Value	Pr > |t|	Lower CL	Upper CL
SVP	Noci-AAb	0.79	0.63	1.26	0.2122	−0.46	2.05
	β2-AAb	−0.72	1.21	−0.60	0.5529	−3.14	1.69
	AT1-AAb	1.24	1.48	0.84	0.4031	−1.70	4.18
	α1-AAb	2.54	0.84	3.01	**0.0035**	0.86	4.22
	MAS-AAb	0.82	1.10	0.74	0.4605	−1.37	3.01
	M2-AAb	1.00	1.19	0.84	0.4038	−1.37	3.37
	ETA-AAb	1.48	1.32	1.12	0.2675	−1.16	4.11
ICP	Noci-AAb	0.38	0.60	0.64	0.5269	−0.81	1.57
	β2-AAb	1.05	0.49	2.15	**0.0344**	0.08	2.01
	AT1-AAb	−0.26	1.05	−0.25	0.8037	−2.36	1.83
	α1-AAb	1.02	0.62	1.64	0.1049	−0.22	2.26
	MAS-AAb	0.41	1.04	0.40	0.6932	−1.66	2.48
	M2-AAb	1.67	1.11	1.50	0.1368	−0.54	3.89
	ETA-AAb	0.06	1.26	0.04	0.9647	−2.44	2.56
DCP	Noci-AAb	0.95	0.67	1.42	0.1610	−0.39	2.30
	β2-AAb	0.35	1.30	0.27	0.7907	−2.25	2.94
	AT1-AAb	2.77	1.16	2.38	**0.0197**	0.45	5.09
	α1-AAb	2.23	0.88	2.52	**0.0138**	0.47	3.98
	MAS-AAb	4.46	1.09	4.10	**0.0001**	2.29	6.63
	M2-AAb	1.02	1.28	0.80	0.4269	−1.52	3.56
	ETA-AAb	0.54	1.43	0.38	0.7055	−2.30	3.39
Peripapillary region	Noci-AAb	−1.51	1.28	−1.18	0.2404	−4.06	1.03
	β2-AAb	−0.86	2.39	−0.36	0.7205	−5.62	3.90
	AT1-AAb	1.40	2.38	0.59	0.5585	−3.35	6.15
	α1-AAb	3.71	1.32	2.82	**0.0062**	1.09	6.34
	MAS-AAb	1.87	2.16	0.86	0.3914	−2.45	6.18
	M2-AAb	0.76	2.35	0.32	0.7487	−3.93	5.45
	ETA-AAb	−3.29	2.98	−1.10	0.2734	−9.24	2.65

## Data Availability

The original contributions presented in the study are included in the article/Supplementary Material.

## Supplementary Materials

The following supporting information can be downloaded at: https://www.mdpi.com/article/10.3390/ijms23137209/s1.
