# Supplementary material for: Long COVID: Association of Functional Autoantibodies against G-Protein-Coupled Receptors with an Impaired Retinal Microcirculation
Source: Int J Mol Sci. 2022 Jun 29;23(13):7209. doi: 10.3390/ijms23137209 (PMC9266742; doi:10.3390/ijms23137209)
Supplement: Supplementary file 1 [file ijms-23-07209-s001.zip › ijms-1769126-supplementary.pdf]

Supplementary material:

Supplementary Table S1 : **Estimations of the age variables (standard error, lower and upper confidence limits, CL) for all the GPCR-AAb on Vessel Density of SVP, ICP, DCP and peripapillary region**. SVP - superficial vascular plexus, ICP - intermediate capillary plexus, DCP - deep capillary plexus. The estimates are represented as the differences between the seronegativity estimate and the seropositivity estimate.

Table S1:

Solution for fixed effects age							
		**Estimate**	**SE**	**t Value**	**Pr > |t|**	**Lower CL**	**Upper CL**
**SVP**	Noci-AAb	-0.04	0.02	-2.17	**0.0330**	-0.07	0.00
	β2-AAb	-0.04	0.02	-2.66	**0.0095**	-0.08	-0.01
	AT1-AAb	-0.07	0.02	-3.32	**0.0014**	-0.12	-0.03
	α1-AAb	-0.07	0.02	-3.26	**0.0017**	-0.11	-0.03
	MAS-AAb	-0.04	0.02	-2.53	**0.0135**	-0.08	-0.01
	M2-AAb	-0.05	0.02	-2.71	**0.0082**	-0.08	-0.01
	ETA-AAb	-0.04	0.02	-2.46	**0.0160**	-0.07	-0.01
**ICP**	Noci-AAb	-0.05	0.02	-2.94	**0.0043**	-0.08	-0.02
	β2-AAb	-0.07	0.01	-10.09	**<.0001**	-0.08	-0.05
	AT1-AAb	-0.05	0.02	-3.18	**0.0021**	-0.08	-0.02
	α1-AAb	-0.05	0.02	-2.97	**0.0039**	-0.08	-0.02
	MAS-AAb	-0.05	0.02	-3.19	**0.0021**	-0.08	-0.02
	M2-AAb	-0.05	0.02	-3.22	**0.0019**	-0.08	-0.02
	ETA-AAb	-0.05	0.02	-3.15	**0.0023**	-0.08	-0.02
**DCP**	Noci-AAb	-0.03	0.02	-1.54	0.1281	-0.06	0.01
	β2-AAb	-0.03	0.02	-1.69	0.0955	-0.07	0.01
	AT1-AAb	-0.02	0.02	-1.27	0.2095	-0.06	0.01
	α1-AAb	-0.05	0.02	-2.03	**0.0452**	-0.09	0.00
	MAS-AAb	-0.02	0.02	-1.28	0.2046	-0.05	0.01
	M2-AAb	-0.03	0.02	-1.81	0.0734	-0.07	0.00
	ETA-AAb	-0.03	0.02	-1.72	0.0897	-0.07	0.00
**Peripapillary region**	Noci-AAb	-0.08	0.03	-2.41	**0.0183**	-0.15	-0.01
	β2-AAb	-0.86	2.39	-0.36	0.7205	-5.62	3.90
	AT1-AAb	1.40	2.38	0.59	0.5585	-3.35	6.15
	α1-AAb	-0.06	0.03	-1.88	0.0638	-0.13	0.00
	MAS-AAb	-0.07	0.03	-2.12	**0.0375**	-0.14	0.00
	M2-AAb	-0.07	0.03	-2.17	**0.0335**	-0.14	-0.01
	ETA-AAb	-0.08	0.03	-2.25	**0.0273**	-0.14	-0.01

Supplementary Table S2: **All the significant differences of the least square means (LS means) of sectorial vessel density (VD) of SVP, ICP, DCP and peripapillary region for**

the significant GPCR-AAb when the interaction term was introduced in the mixed model. The p-values of the multiple comparisons are adjusted after Tukey-Kramer Test. SVP - superficial vascular plexus, ICP - intermediate capillary plexus, DCP - deep capillary plexus.

Table S2:

Differences of Least Squares Means (Sector*M2-AAb) for SVP**													
**Effect**	**Sector**	**M2-AAb***	**_Sector**	**_M2-AAb***	**Estimate**	**SE**	**t Value**	**Pr > |t|**	**Adj P**	**Lower**	**Upper**	**Adj Lower**	**Adj Upper**
**M2-AAb**		0		1	1.39	1.60	0.87	0.3895	0.3895	-1.81	4.58	-1.81	4.58
**M2-AAb*Sector**	sector10	1	sector6	1	-1.90	0.35	-5.47	<.0001	0.0001	-2.59	-1.21	-3.21	-0.59
**M2-AAb*Sector**	sector4	1	sector9	1	1.89	0.35	5.43	<.0001	0.0001	1.20	2.58	0.58	3.20
**M2-AAb*Sector**	sector12	1	sector9	1	1.53	0.29	5.24	<.0001	0.0003	0.95	2.11	0.43	2.63
**M2-AAb*Sector**	sector5	1	sector9	1	1.60	0.31	5.14	<.0001	0.0005	0.98	2.22	0.43	2.77
**M2-AAb*Sector**	sector10	1	sector4	1	-1.68	0.33	-5.03	<.0001	0.0007	-2.35	-1.02	-2.94	-0.42
**M2-AAb*Sector**	sector1	1	sector10	1	1.61	0.34	4.75	<.0001	0.002	0.94	2.29	0.33	2.90
**M2-AAb*Sector**	sector10	1	sector7	1	-1.68	0.36	-4.68	<.0001	0.0026	-2.40	-0.97	-3.04	-0.33
**M2-AAb*Sector**	sector3	1	sector9	1	1.49	0.32	4.63	<.0001	0.0031	0.85	2.13	0.28	2.71
**M2-AAb*Sector**	sector2	1	sector9	1	1.55	0.34	4.6	<.0001	0.0035	0.88	2.22	0.28	2.82
**M2-AAb*Sector**	sector8	1	sector9	1	1.31	0.29	4.56	<.0001	0.004	0.74	1.88	0.23	2.39
**M2-AAb*Sector**	sector10	1	sector5	1	-1.39	0.31	-4.46	<.0001	0.0057	-2.01	-0.77	-2.57	-0.21
**M2-AAb*Sector**	sector10	1	sector12	1	-1.32	0.31	-4.22	<.0001	0.0125	-1.94	-0.70	-2.50	-0.14
**M2-AAb*Sector**	sector10	1	sector3	1	-1.29	0.31	-4.17	<.0001	0.0149	-1.90	-0.67	-2.45	-0.12
**M2-AAb*Sector**	sector11	1	sector9	1	1.18	0.30	3.9	0.0002	0.0347	0.58	1.78	0.04	2.32
**M2-AAb*Sector**	sector10	1	sector2	1	-1.34	0.36	-3.72	0.0004	0.0577	-2.06	-0.63	-2.71	0.02
**M2-AAb*Sector**	sector10	1	sector11	1	-0.97	0.27	-3.56	0.0006	0.0897	-1.52	-0.43	-2.01	0.06

**M2-AAb*Sector**	sector5	0	sector7	0	-3.24	0.98	-3.29	0.0015	0.1749	-5.20	-1.28	-6.95	0.47
**M2-AAb*Sector**	sector7	0	sector9	0	4.34	1.32	3.28	0.0016	0.1798	1.70	6.97	-0.65	9.33
**M2-AAb*Sector**	sector10	1	sector8	1	-1.10	0.34	-3.23	0.0018	0.1986	-1.78	-0.42	-2.39	0.18
**M2-AAb*Sector**	sector4	0	sector7	0	-3.71	1.27	-2.92	0.0045	0.368	-6.24	-1.19	-8.50	1.07
**M2-AAb*Sector**	sector6	1	sector8	1	0.80	0.28	2.9	0.0049	0.3868	0.25	1.35	-0.24	1.84
**M2-AAb*Sector**	sector10	0	sector7	0	-3.92	1.41	-2.78	0.0069	0.4702	-6.73	-1.11	-9.24	1.40
**M2-AAb*Sector**	sector11	1	sector6	1	-0.93	0.35	-2.67	0.0091	0.5448	-1.62	-0.24	-2.23	0.38
**M2-AAb*Sector**	sector7	0	sector9	1	5.12	1.95	2.63	0.0102	0.5764	1.25	8.99	-2.22	12.45
**M2-AAb*Sector**	sector7	0	sector10	1	4.91	1.95	2.52	0.0137	0.6559	1.04	8.79	-2.43	12.25
**M2-AAb*Sector**	sector2	0	sector9	0	3.25	1.32	2.45	0.0164	0.7055	0.61	5.88	-1.74	8.24
**M2-AAb*Sector**	sector11	1	sector4	1	-0.71	0.29	-2.45	0.0165	0.7063	-1.28	-0.13	-1.80	0.38
**M2-AAb*Sector**	sector7	1	sector8	1	0.58	0.24	2.43	0.0176	0.7242	0.10	1.06	-0.32	1.48
**M2-AAb*Sector**	sector2	0	sector9	1	4.03	1.72	2.34	0.0217	0.778	0.60	7.45	-2.46	10.51
**M2-AAb*Sector**	sector7	0	sector8	0	2.17	0.94	2.32	0.0231	0.7938	0.31	4.04	-1.36	5.71
**M2-AAb*Sector**	sector12	0	sector9	0	2.60	1.14	2.28	0.0255	0.8163	0.33	4.88	-1.71	6.91
**M2-AAb*Sector**	sector6	0	sector9	0	3.15	1.41	2.24	0.0281	0.8386	0.35	5.96	-2.16	8.47
**M2-AAb*Sector**	sector2	0	sector10	1	3.82	1.72	2.22	0.0293	0.8477	0.39	7.25	-2.67	10.31
**M2-AAb*Sector**	sector2	0	sector4	0	2.62	1.19	2.21	0.0301	0.853	0.26	4.98	-1.85	7.10

**M2-AAb*Sector**	sector1	1	sector11	1	0.64	0.30	2.15	0.0343	0.8794	0.05	1.23	-0.48	1.76
**M2-AAb*Sector**	sector4	0	sector6	0	-2.53	1.18	-2.15	0.0347	0.8813	-4.87	-0.19	-6.96	1.91
**M2-AAb*Sector**	sector11	1	sector7	1	-0.71	0.34	-2.06	0.0431	0.9183	-1.39	-0.02	-2.01	0.59
**M2-AAb*Sector**	sector7	0	sector11	1	3.94	1.92	2.05	0.0437	0.9206	0.11	7.76	-3.31	11.18
**M2-AAb*Sector**	sector3	1	sector6	1	-0.62	0.31	-2.02	0.0468	0.9305	-1.22	-0.01	-1.77	0.53
**M2-AAb*Sector**	sector10	0	sector6	0	-2.73	1.36	-2	0.0486	0.9355	-5.45	-0.02	-7.88	2.41
**M2-AAb*Sector**	sector10	0	sector2	0	-2.83	1.42	-1.99	0.0497	0.9383	-5.65	0.00	-8.18	2.52
**M2-AAb*Sector**	sector6	0	sector9	1	3.93	1.97	1.99	0.0497	0.9384	0.00	7.86	-3.51	11.38
**M2-AAb*Sector**	sector1	1	sector9	1	1.82	0.32	5.67	<.0001	<.0001	1.18	2.46	0.61	3.03
**M2-AAb*Sector**	sector6	1	sector9	1	2.11	0.36	5.87	<.0001	<.0001	1.39	2.82	0.75	3.46
**M2-AAb*Sector**	sector7	1	sector9	1	1.89	0.34	5.6	<.0001	<.0001	1.22	2.56	0.62	3.16
**M2-AAb*Sector**	sector7	1	sector9	1	1.89	0.34	5.6	<.0001	<.0001	1.22	2.56	0.62	3.16

** *interaction term significant p-value=0.038*

**Differences of Least Squares Means (Sector*Noci-AAb) for ICP****													
**Effect**	**Sector**	**Noci-AAb***	**_Sector**	**_Noci-AAb***	**Estimate**	**SE**	**t Value**	**Pr > |t|**	**Adj P**	**Lower**	**Upper**	**Adj Lower**	**Adj Upper**
**Noci-AAb**		0		1	-0.03	0.74	-0.04	0.9685	0.9685	-1.51	1.45	-1.51	1.45
**Noci-AAb*Sector**	sector10	0	sector5	0	1.91	0.37	5.11	<.0001	0.0001	1.18	2.64	0.55	3.27
**Noci-AAb*Sector**	sector1	0	sector3	0	-1.87	0.37	-5.02	<.0001	0.0002	-2.61	-1.14	-3.23	-0.51
**Noci-AAb*Sector**	sector1	0	sector4	0	-1.87	0.37	-5	<.0001	0.0002	-2.60	-1.13	-3.23	-0.51

**Noci-AAb*Sector**	sector11	0	sector7	0	1.82	0.37	4.87	<.0001	0.0003	1.09	2.55	0.46	3.18
**Noci-AAb*Sector**	sector12	1	sector4	1	-2.68	0.55	-4.89	<.0001	0.0003	-3.75	-1.60	-4.68	-0.68
**Noci-AAb*Sector**	sector12	0	sector3	0	-1.76	0.37	-4.71	<.0001	0.0007	-2.49	-1.02	-3.12	-0.40
**Noci-AAb*Sector**	sector12	0	sector4	0	-1.75	0.37	-4.69	<.0001	0.0008	-2.48	-1.02	-3.11	-0.39
**Noci-AAb*Sector**	sector6	0	sector9	0	-1.73	0.37	-4.63	<.0001	0.001	-2.46	-1.00	-3.09	-0.37
**Noci-AAb*Sector**	sector10	1	sector12	1	2.46	0.55	4.49	<.0001	0.0019	1.39	3.54	0.46	4.46
**Noci-AAb*Sector**	sector1	0	sector10	0	-1.66	0.37	-4.46	<.0001	0.0022	-2.40	-0.93	-3.03	-0.30
**Noci-AAb*Sector**	sector1	1	sector4	1	-2.40	0.55	-4.38	<.0001	0.0032	-3.47	-1.32	-4.40	-0.40
**Noci-AAb*Sector**	sector2	0	sector7	0	1.61	0.37	4.31	<.0001	0.0042	0.88	2.34	0.25	2.97
**Noci-AAb*Sector**	sector10	0	sector12	0	1.55	0.37	4.15	<.0001	0.008	0.82	2.28	0.19	2.91
**Noci-AAb*Sector**	sector11	0	sector6	0	1.53	0.37	4.11	<.0001	0.0094	0.80	2.27	0.17	2.90
**Noci-AAb*Sector**	sector12	1	sector3	1	-2.21	0.55	-4.04	<.0001	0.0125	-3.29	-1.14	-4.21	-0.21
**Noci-AAb*Sector**	sector8	0	sector9	0	-1.49	0.37	-3.98	<.0001	0.0156	-2.22	-0.75	-2.85	-0.12
**Noci-AAb*Sector**	sector1	1	sector10	1	-2.18	0.55	-3.98	<.0001	0.0158	-3.25	-1.10	-4.18	-0.18
**Noci-AAb*Sector**	sector5	0	sector9	0	-1.38	0.37	-3.7	0.0002	0.0417	-2.11	-0.65	-2.74	-0.02
**Noci-AAb*Sector**	sector4	1	sector6	1	1.95	0.55	3.56	0.0004	0.0672	0.87	3.02	-0.05	3.95
**Noci-AAb*Sector**	sector2	0	sector6	0	1.33	0.37	3.55	0.0004	0.0683	0.59	2.06	-0.04	2.69
**Noci-AAb*Sector**	sector11	1	sector4	1	-1.94	0.55	-3.53	0.0004	0.072	-3.01	-0.86	-3.93	0.06

**Noci-AAb*Sector**	sector1	1	sector3	1	-1.93	0.55	-3.52	0.0005	0.0749	-3.00	-0.85	-3.93	0.07
**Noci-AAb*Sector**	sector11	0	sector8	0	1.29	0.37	3.46	0.0006	0.0907	0.56	2.02	-0.07	2.65
**Noci-AAb*Sector**	sector7	0	sector4	1	-2.84	0.87	-3.26	0.0011	0.1579	-4.54	-1.13	-6.01	0.34
**Noci-AAb*Sector**	sector2	1	sector4	1	-1.79	0.55	-3.26	0.0012	0.1587	-2.86	-0.71	-3.78	0.21
**Noci-AAb*Sector**	sector11	0	sector5	0	1.19	0.37	3.18	0.0015	0.1938	0.46	1.92	-0.17	2.55
**Noci-AAb*Sector**	sector12	1	sector9	1	-1.73	0.55	-3.17	0.0016	0.2015	-2.81	-0.66	-3.73	0.26
**Noci-AAb*Sector**	sector10	1	sector6	1	1.73	0.55	3.16	0.0016	0.2058	0.65	2.81	-0.27	3.73
**Noci-AAb*Sector**	sector10	1	sector11	1	1.72	0.55	3.14	0.0018	0.2173	0.64	2.79	-0.28	3.72
**Noci-AAb*Sector**	sector4	1	sector7	1	1.68	0.55	3.06	0.0023	0.26	0.60	2.75	-0.32	3.67
**Noci-AAb*Sector**	sector1	0	sector9	0	-1.14	0.37	-3.05	0.0024	0.2658	-1.87	-0.41	-2.50	0.22
**Noci-AAb*Sector**	sector2	0	sector3	0	-1.14	0.37	-3.05	0.0024	0.2667	-1.87	-0.41	-2.50	0.22
**Noci-AAb*Sector**	sector2	0	sector4	0	-1.13	0.37	-3.03	0.0025	0.2757	-1.86	-0.40	-2.49	0.23
**Noci-AAb*Sector**	sector7	0	sector10	1	-2.62	0.87	-3.01	0.0027	0.2896	-4.33	-0.91	-5.79	0.55
**Noci-AAb*Sector**	sector3	0	sector12	1	2.59	0.87	2.98	0.003	0.3105	0.88	4.30	-0.58	5.76
**Noci-AAb*Sector**	sector4	0	sector12	1	2.58	0.87	2.97	0.003	0.3147	0.88	4.29	-0.59	5.76
**Noci-AAb*Sector**	sector6	0	sector4	1	-2.55	0.87	-2.94	0.0034	0.3393	-4.26	-0.85	-5.73	0.62
**Noci-AAb*Sector**	sector2	0	sector8	0	1.08	0.37	2.9	0.0038	0.3647	0.35	1.81	-0.28	2.44
**Noci-AAb*Sector**	sector10	1	sector2	1	1.57	0.55	2.86	0.0043	0.3923	0.49	2.64	-0.43	3.57

**Noci-AAb*Sector**	sector12	0	sector9	0	-1.02	0.37	-2.74	0.0063	0.4849	-1.76	-0.29	-2.38	0.34
**Noci-AAb*Sector**	sector10	0	sector12	1	2.38	0.87	2.74	0.0063	0.4858	0.68	4.09	-0.79	5.56
**Noci-AAb*Sector**	sector7	0	sector3	1	-2.37	0.87	-2.72	0.0066	0.4974	-4.08	-0.66	-5.54	0.80
**Noci-AAb*Sector**	sector3	1	sector6	1	1.48	0.55	2.7	0.007	0.5139	0.41	2.56	-0.52	3.48
**Noci-AAb*Sector**	sector4	1	sector5	1	1.48	0.55	2.7	0.0072	0.5206	0.40	2.55	-0.52	3.48
**Noci-AAb*Sector**	sector6	0	sector10	1	-2.33	0.87	-2.68	0.0074	0.5296	-4.04	-0.63	-5.51	0.84
**Noci-AAb*Sector**	sector11	1	sector3	1	-1.47	0.55	-2.68	0.0075	0.5318	-2.54	-0.39	-3.47	0.53
**Noci-AAb*Sector**	sector10	1	sector7	1	1.46	0.55	2.66	0.0079	0.5487	0.38	2.53	-0.54	3.46
**Noci-AAb*Sector**	sector8	0	sector4	1	-2.31	0.87	-2.66	0.0081	0.5527	-4.02	-0.60	-5.48	0.86
**Noci-AAb*Sector**	sector12	0	sector7	0	0.99	0.37	2.65	0.0081	0.5548	0.26	1.72	-0.37	2.35
**Noci-AAb*Sector**	sector3	0	sector1	1	2.31	0.87	2.65	0.0081	0.5549	0.60	4.01	-0.87	5.48
**Noci-AAb*Sector**	sector1	1	sector9	1	-1.45	0.55	-2.65	0.0082	0.5572	-2.53	-0.38	-3.45	0.55
**Noci-AAb*Sector**	sector4	0	sector1	1	2.30	0.87	2.65	0.0083	0.56	0.59	4.01	-0.87	5.47
**Noci-AAb*Sector**	sector2	0	sector5	0	0.98	0.37	2.62	0.0089	0.579	0.25	1.71	-0.38	2.34
**Noci-AAb*Sector**	sector5	0	sector4	1	-2.21	0.87	-2.54	0.0114	0.6472	-3.91	-0.50	-5.38	0.97
**Noci-AAb*Sector**	sector1	0	sector11	0	-0.94	0.37	-2.53	0.0116	0.6537	-1.68	-0.21	-2.31	0.42
**Noci-AAb*Sector**	sector4	1	sector8	1	1.38	0.55	2.52	0.0119	0.6592	0.31	2.46	-0.62	3.38
**Noci-AAb*Sector**	sector10	0	sector2	0	0.93	0.37	2.49	0.0129	0.6824	0.20	1.66	-0.43	2.29

**Noci-AAb*Sector**	sector11	0	sector3	0	-0.93	0.37	-2.49	0.013	0.6846	-1.66	-0.20	-2.29	0.43
**Noci-AAb*Sector**	sector11	0	sector4	0	-0.92	0.37	-2.47	0.0136	0.6958	-1.66	-0.19	-2.29	0.44
**Noci-AAb*Sector**	sector10	0	sector1	1	2.10	0.87	2.41	0.016	0.7394	0.39	3.81	-1.07	5.27
**Noci-AAb*Sector**	sector2	1	sector3	1	-1.32	0.55	-2.41	0.0163	0.7442	-2.39	-0.24	-3.32	0.68
**Noci-AAb*Sector**	sector8	0	sector10	1	-2.09	0.87	-2.4	0.0164	0.7461	-3.80	-0.38	-5.26	1.08
**Noci-AAb*Sector**	sector6	0	sector3	1	-2.09	0.87	-2.4	0.0167	0.7503	-3.79	-0.38	-5.26	1.09
**Noci-AAb*Sector**	sector12	1	sector8	1	-1.30	0.55	-2.37	0.018	0.7693	-2.37	-0.22	-3.30	0.70
**Noci-AAb*Sector**	sector1	0	sector7	0	0.87	0.37	2.34	0.0193	0.7869	0.14	1.61	-0.49	2.24
**Noci-AAb*Sector**	sector10	1	sector5	1	1.26	0.55	2.3	0.0219	0.8164	0.18	2.33	-0.74	3.26
**Noci-AAb*Sector**	sector5	0	sector10	1	-1.99	0.87	-2.29	0.0225	0.8231	-3.69	-0.28	-5.16	1.19
**Noci-AAb*Sector**	sector1	0	sector4	1	-1.96	0.87	-2.26	0.0243	0.84	-3.67	-0.26	-5.14	1.21
**Noci-AAb*Sector**	sector11	0	sector12	0	0.83	0.37	2.22	0.0267	0.8597	0.10	1.56	-0.53	2.19
**Noci-AAb*Sector**	sector3	1	sector7	1	1.21	0.55	2.21	0.0276	0.8665	0.13	2.28	-0.79	3.21
**Noci-AAb*Sector**	sector12	1	sector5	1	-1.20	0.55	-2.2	0.0283	0.8714	-2.28	-0.13	-3.20	0.80
**Noci-AAb*Sector**	sector7	0	sector9	1	-1.89	0.87	-2.18	0.0299	0.8814	-3.60	-0.19	-5.07	1.28
**Noci-AAb*Sector**	sector3	0	sector6	1	1.86	0.87	2.14	0.0328	0.8983	0.15	3.57	-1.31	5.03
**Noci-AAb*Sector**	sector9	0	sector12	1	1.86	0.87	2.13	0.0331	0.8998	0.15	3.56	-1.32	5.03
**Noci-AAb*Sector**	sector4	0	sector6	1	1.85	0.87	2.13	0.0333	0.9009	0.15	3.56	-1.32	5.03

**Noci-AAb*Sector**	sector12	0	sector4	1	-1.85	0.87	-2.12	0.034	0.9041	-3.55	-0.14	-5.02	1.33
**Noci-AAb*Sector**	sector3	0	sector11	1	1.85	0.87	2.12	0.034	0.9042	0.14	3.55	-1.33	5.02
**Noci-AAb*Sector**	sector10	1	sector8	1	1.16	0.55	2.12	0.0341	0.9045	0.09	2.24	-0.84	3.16
**Noci-AAb*Sector**	sector8	0	sector3	1	-1.84	0.87	-2.12	0.0344	0.9063	-3.55	-0.14	-5.02	1.33
**Noci-AAb*Sector**	sector4	0	sector11	1	1.84	0.87	2.12	0.0345	0.9067	0.13	3.55	-1.33	5.01
**Noci-AAb*Sector**	sector1	0	sector10	1	-1.74	0.87	-2	0.0453	0.9447	-3.45	-0.04	-4.92	1.43
**Noci-AAb*Sector**	sector5	0	sector3	1	-1.74	0.87	-2	0.0459	0.9462	-3.45	-0.03	-4.91	1.43
**Noci-AAb*Sector**	sector1	0	sector2	0	-0.73	0.37	-1.97	0.0493	0.9541	-1.47	0.00	-2.10	0.63
**Noci-AAb*Sector**	sector3	0	sector9	0	0.73	0.37	1.97	0.0495	0.9545	0.00	1.47	-0.63	2.10
**Noci-AAb*Sector**	sector10	0	sector6	0	2.26	0.37	6.04	<.0001	<.0001	1.52	2.99	0.89	3.62
**Noci-AAb*Sector**	sector10	0	sector7	0	2.54	0.37	6.8	<.0001	<.0001	1.81	3.27	1.18	3.90
**Noci-AAb*Sector**	sector10	0	sector8	0	2.01	0.37	5.39	<.0001	<.0001	1.28	2.74	0.65	3.37
**Noci-AAb*Sector**	sector3	0	sector5	0	2.12	0.37	5.67	<.0001	<.0001	1.38	2.85	0.75	3.48
**Noci-AAb*Sector**	sector3	0	sector6	0	2.46	0.37	6.6	<.0001	<.0001	1.73	3.20	1.10	3.83
**Noci-AAb*Sector**	sector3	0	sector7	0	2.75	0.37	7.36	<.0001	<.0001	2.01	3.48	1.39	4.11
**Noci-AAb*Sector**	sector3	0	sector8	0	2.22	0.37	5.95	<.0001	<.0001	1.49	2.95	0.86	3.58
**Noci-AAb*Sector**	sector4	0	sector5	0	2.11	0.37	5.66	<.0001	<.0001	1.38	2.84	0.75	3.47
**Noci-AAb*Sector**	sector4	0	sector6	0	2.46	0.37	6.59	<.0001	<.0001	1.73	3.19	1.10	3.82

**Noci-AAb*Sector**	sector4	0	sector7	0	2.74	0.37	7.35	<.0001	<.0001	2.01	3.47	1.38	4.10
**Noci-AAb*Sector**	sector4	0	sector8	0	2.21	0.37	5.93	<.0001	<.0001	1.48	2.95	0.85	3.58
**Noci-AAb*Sector**	sector7	0	sector9	0	-2.01	0.37	-5.39	<.0001	<.0001	-2.75	-1.28	-3.38	-0.65
**Noci-AAb*Sector**	sector7	0	sector9	0	-2.01	0.37	-5.39	<.0001	<.0001	-2.75	-1.28	-3.38	-0.65

*** interaction term significant p-value=0.013*

**Differences of Least Squares Means (Sector*Noci-AAb) for DCP****													
**Effect**	**Sector**	**Noci-AAb***	**_Sector**	**_Noci-AAb***	**Estimate**	**SE**	**t Value**	**Pr > |t|**	**Adj P**	**Lower**	**Upper**	**Adj Lower**	**Adj Upper**
**Noci-AAb**		0		1	0.37	0.86	0.42	0.6732	0.6732	-1.35	2.09	-1.35	2.09
**Noci-AAb*Sector**	sector4	0	sector6	0	2.01	0.37	5.44	<.0001	0.0001	1.27	2.74	0.62	3.40
**Noci-AAb*Sector**	sector3	0	sector6	0	2.39	0.45	5.36	<.0001	0.0002	1.50	3.28	0.71	4.07
**Noci-AAb*Sector**	sector3	0	sector5	0	2.09	0.39	5.29	<.0001	0.0003	1.30	2.88	0.60	3.58
**Noci-AAb*Sector**	sector3	0	sector9	0	2.43	0.49	4.92	<.0001	0.0011	1.45	3.41	0.57	4.29
**Noci-AAb*Sector**	sector2	0	sector8	0	2.29	0.51	4.46	<.0001	0.0055	1.27	3.31	0.36	4.23
**Noci-AAb*Sector**	sector10	0	sector8	0	1.75	0.42	4.12	<.0001	0.0173	0.90	2.59	0.15	3.34
**Noci-AAb*Sector**	sector4	0	sector9	0	2.04	0.51	4.02	0.0001	0.0237	1.03	3.05	0.13	3.96
**Noci-AAb*Sector**	sector11	0	sector3	0	-1.51	0.47	-3.23	0.0018	0.2016	-2.44	-0.58	-3.27	0.25
**Noci-AAb*Sector**	sector11	1	sector4	1	-2.49	0.78	-3.2	0.002	0.2119	-4.04	-0.94	-5.42	0.44
**Noci-AAb*Sector**	sector2	0	sector7	0	1.73	0.54	3.2	0.002	0.2144	0.65	2.81	-0.31	3.78
**Noci-AAb*Sector**	sector12	1	sector4	1	-2.57	0.81	-3.18	0.0021	0.2204	-4.17	-0.96	-5.61	0.47

**Noci-AAb*Sector**	sector10	0	sector3	0	-1.37	0.43	-3.18	0.0021	0.2218	-2.22	-0.51	-2.98	0.25
**Noci-AAb*Sector**	sector3	0	sector12	1	3.17	1.03	3.09	0.0028	0.2677	1.13	5.22	-0.70	7.04
**Noci-AAb*Sector**	sector2	0	sector9	0	1.61	0.53	3.06	0.0031	0.2879	0.56	2.65	-0.38	3.59
**Noci-AAb*Sector**	sector12	0	sector8	0	1.51	0.51	2.98	0.0038	0.3328	0.50	2.52	-0.40	3.43
**Noci-AAb*Sector**	sector2	0	sector6	0	1.57	0.53	2.95	0.0042	0.3508	0.51	2.63	-0.44	3.58
**Noci-AAb*Sector**	sector12	1	sector2	1	-1.61	0.55	-2.92	0.0046	0.3721	-2.71	-0.51	-3.69	0.47
**Noci-AAb*Sector**	sector4	1	sector5	1	1.26	0.43	2.89	0.0049	0.388	0.39	2.12	-0.38	2.89
**Noci-AAb*Sector**	sector12	1	sector3	1	-2.03	0.71	-2.88	0.0052	0.3997	-3.43	-0.62	-4.69	0.63
**Noci-AAb*Sector**	sector1	0	sector8	0	1.62	0.57	2.87	0.0052	0.4013	0.50	2.75	-0.51	3.75
**Noci-AAb*Sector**	sector11	1	sector3	1	-1.95	0.69	-2.84	0.0057	0.423	-3.31	-0.58	-4.54	0.64
**Noci-AAb*Sector**	sector4	0	sector11	1	2.71	0.96	2.81	0.0063	0.4457	0.79	4.63	-0.93	6.35
**Noci-AAb*Sector**	sector5	0	sector8	0	1.02	0.37	2.8	0.0065	0.4545	0.29	1.75	-0.36	2.40
**Noci-AAb*Sector**	sector8	0	sector4	1	-2.51	0.93	-2.7	0.0084	0.522	-4.35	-0.66	-6.00	0.99
**Noci-AAb*Sector**	sector1	1	sector4	1	-2.12	0.80	-2.66	0.0094	0.5526	-3.71	-0.54	-5.12	0.88
**Noci-AAb*Sector**	sector4	0	sector12	1	2.79	1.05	2.66	0.0095	0.5572	0.70	4.88	-1.17	6.75
**Noci-AAb*Sector**	sector11	1	sector2	1	-1.53	0.58	-2.63	0.0104	0.5798	-2.69	-0.37	-3.73	0.67
**Noci-AAb*Sector**	sector10	1	sector4	1	-1.73	0.66	-2.62	0.0104	0.5814	-3.04	-0.42	-4.21	0.75
**Noci-AAb*Sector**	sector2	0	sector5	0	1.27	0.49	2.58	0.0117	0.6119	0.29	2.25	-0.58	3.12

**Noci-AAb*Sector**	sector4	1	sector9	1	1.90	0.75	2.55	0.0127	0.6361	0.42	3.39	-0.91	4.71
**Noci-AAb*Sector**	sector3	0	sector1	1	2.73	1.07	2.54	0.0132	0.6463	0.59	4.86	-1.33	6.78
**Noci-AAb*Sector**	sector3	0	sector10	1	2.33	0.93	2.5	0.0145	0.6723	0.48	4.19	-1.19	5.85
**Noci-AAb*Sector**	sector3	0	sector9	1	2.51	1.02	2.46	0.016	0.6982	0.48	4.53	-1.33	6.34
**Noci-AAb*Sector**	sector2	0	sector3	0	-0.82	0.34	-2.41	0.0185	0.7371	-1.50	-0.14	-2.11	0.47
**Noci-AAb*Sector**	sector1	1	sector3	1	-1.58	0.66	-2.39	0.0192	0.7467	-2.90	-0.26	-4.07	0.91
**Noci-AAb*Sector**	sector10	0	sector7	0	1.19	0.50	2.39	0.0192	0.7476	0.20	2.18	-0.69	3.06
**Noci-AAb*Sector**	sector1	1	sector2	1	-1.16	0.50	-2.31	0.0233	0.7952	-2.16	-0.16	-3.05	0.73
**Noci-AAb*Sector**	sector6	0	sector8	0	0.72	0.32	2.29	0.025	0.8118	0.09	1.35	-0.47	1.91
**Noci-AAb*Sector**	sector2	0	sector11	1	2.27	1.00	2.28	0.0255	0.8171	0.29	4.26	-1.49	6.04
**Noci-AAb*Sector**	sector8	0	sector3	1	-1.97	0.87	-2.26	0.0264	0.8249	-3.70	-0.24	-5.24	1.31
**Noci-AAb*Sector**	sector4	1	sector6	1	1.20	0.54	2.22	0.0294	0.8485	0.12	2.28	-0.84	3.24
**Noci-AAb*Sector**	sector12	0	sector4	0	-1.21	0.55	-2.21	0.0301	0.8532	-2.31	-0.12	-3.29	0.86
**Noci-AAb*Sector**	sector10	0	sector4	0	-0.98	0.45	-2.19	0.0319	0.865	-1.87	-0.09	-2.67	0.71
**Noci-AAb*Sector**	sector2	0	sector12	1	2.35	1.08	2.18	0.0326	0.8694	0.20	4.51	-1.72	6.43
**Noci-AAb*Sector**	sector10	0	sector6	0	1.03	0.48	2.16	0.0341	0.8782	0.08	1.97	-0.77	2.82
**Noci-AAb*Sector**	sector11	0	sector9	0	0.92	0.43	2.15	0.0344	0.88	0.07	1.77	-0.69	2.53
**Noci-AAb*Sector**	sector4	0	sector1	1	2.34	1.10	2.13	0.036	0.8881	0.16	4.52	-1.79	6.48

**Noci-AAb*Sector**	sector11	0	sector4	0	-1.12	0.53	-2.12	0.0372	0.8943	-2.18	-0.07	-3.12	0.87
**Noci-AAb*Sector**	sector12	0	sector2	0	-0.78	0.38	-2.07	0.0417	0.9132	-1.53	-0.03	-2.20	0.64
**Noci-AAb*Sector**	sector7	0	sector4	1	-1.95	0.95	-2.05	0.0434	0.9196	-3.84	-0.06	-5.53	1.63
**Noci-AAb*Sector**	sector4	0	sector9	1	2.12	1.04	2.04	0.0449	0.9244	0.05	4.19	-1.80	6.05
**Noci-AAb*Sector**	sector1	0	sector4	0	-1.10	0.54	-2.03	0.0454	0.926	-2.18	-0.02	-3.15	0.94
**Noci-AAb*Sector**	sector4	0	sector10	1	1.95	0.96	2.03	0.0455	0.9263	0.04	3.86	-1.67	5.56
**Noci-AAb*Sector**	sector3	0	sector5	1	1.86	0.92	2.02	0.0473	0.9317	0.02	3.70	-1.62	5.34
**Noci-AAb*Sector**	sector3	0	sector7	0	2.55	0.44	5.77	<.0001	<.0001	1.67	3.43	0.89	4.22
**Noci-AAb*Sector**	sector3	0	sector8	0	3.11	0.43	7.2	<.0001	<.0001	2.25	3.97	1.48	4.74
**Noci-AAb*Sector**	sector4	0	sector5	0	1.71	0.30	5.77	<.0001	<.0001	1.12	2.29	0.59	2.82
**Noci-AAb*Sector**	sector4	0	sector7	0	2.17	0.38	5.64	<.0001	<.0001	1.40	2.93	0.72	3.62
**Noci-AAb*Sector**	sector4	0	sector8	0	2.73	0.45	6.05	<.0001	<.0001	1.83	3.62	1.03	4.43

** *interaction term significant p-value=0.034*

**Differences of Least Squares Means (Sector*β2-AAb) for DCP****													
**Effect**	**Sector**	**β2-AAb***	**_Sector**	**_β2-AAb***	**Estimate**	**SE**	**t Value**	**Pr > |t|**	**Adj P**	**Lower**	**Upper**	**Adj Lower**	**Adj Upper**
**β2-AAb**		0		1	1.43	1.66	0.87	0.3896	0.3896	-1.86	4.73	-1.86	4.73
**β2-AAb*Sector**	sector3	1	sector6	1	1.90	0.39	4.85	<.0001	0.0014	1.12	2.67	0.42	3.37
**β2-AAb*Sector**	sector3	1	sector5	1	1.65	0.34	4.79	<.0001	0.0017	0.97	2.34	0.35	2.95
**β2-AAb*Sector**	sector3	1	sector7	1	1.84	0.39	4.69	<.0001	0.0025	1.06	2.62	0.36	3.32

**β2-AAb*Sector**	sector11	1	sector3	1	-1.79	0.39	-4.53	<.0001	0.0043	-2.57	-1.00	-3.27	-0.30
**β2-AAb*Sector**	sector12	1	sector3	1	-1.82	0.41	-4.46	<.0001	0.0057	-2.63	-1.01	-3.36	-0.28
**β2-AAb*Sector**	sector1	1	sector3	1	-1.57	0.38	-4.09	0.0001	0.019	-2.33	-0.80	-3.01	-0.12
**β2-AAb*Sector**	sector11	1	sector4	1	-1.80	0.44	-4.04	0.0001	0.0221	-2.68	-0.91	-3.47	-0.12
**β2-AAb*Sector**	sector10	1	sector3	1	-1.44	0.36	-3.97	0.0002	0.0274	-2.16	-0.72	-2.80	-0.07
**β2-AAb*Sector**	sector12	1	sector4	1	-1.83	0.47	-3.92	0.0002	0.0321	-2.76	-0.90	-3.59	-0.07
**β2-AAb*Sector**	sector10	1	sector4	1	-1.45	0.37	-3.9	0.0002	0.0343	-2.18	-0.71	-2.84	-0.05
**β2-AAb*Sector**	sector2	1	sector8	1	1.59	0.45	3.52	0.0007	0.0981	0.69	2.49	-0.11	3.30
**β2-AAb*Sector**	sector1	1	sector4	1	-1.58	0.46	-3.42	0.001	0.1267	-2.49	-0.66	-3.31	0.16
**β2-AAb*Sector**	sector2	1	sector9	1	1.48	0.45	3.31	0.0014	0.1645	0.59	2.38	-0.20	3.17
**β2-AAb*Sector**	sector10	1	sector9	1	0.86	0.26	3.27	0.0016	0.1824	0.34	1.39	-0.13	1.86
**β2-AAb*Sector**	sector12	1	sector2	1	-1.01	0.32	-3.11	0.0026	0.257	-1.65	-0.36	-2.22	0.21
**β2-AAb*Sector**	sector11	1	sector2	1	-0.97	0.34	-2.85	0.0056	0.4187	-1.65	-0.29	-2.26	0.31
**β2-AAb*Sector**	sector2	1	sector3	1	-0.81	0.29	-2.84	0.0057	0.4217	-1.38	-0.24	-1.89	0.27
**β2-AAb*Sector**	sector7	0	sector9	0	-3.79	1.46	-2.6	0.0113	0.6023	-6.70	-0.88	-9.30	1.72
**β2-AAb*Sector**	sector1	1	sector2	1	-0.75	0.29	-2.59	0.0115	0.6091	-1.33	-0.17	-1.85	0.34
**β2-AAb*Sector**	sector10	1	sector8	1	0.97	0.38	2.54	0.0131	0.6437	0.21	1.73	-0.47	2.41
**β2-AAb*Sector**	sector7	1	sector8	1	0.57	0.23	2.45	0.0165	0.7065	0.11	1.03	-0.30	1.44

**β2-AAb*Sector**	sector5	1	sector8	1	0.76	0.31	2.41	0.0183	0.7342	0.13	1.38	-0.43	1.94
**β2-AAb*Sector**	sector2	1	sector6	1	1.08	0.46	2.36	0.0209	0.7686	0.17	1.99	-0.65	2.81
**β2-AAb*Sector**	sector2	1	sector4	1	-0.82	0.37	-2.25	0.0274	0.8327	-1.55	-0.09	-2.21	0.56
**β2-AAb*Sector**	sector2	1	sector7	1	1.03	0.46	2.21	0.0299	0.852	0.10	1.95	-0.72	2.78
**β2-AAb*Sector**	sector7	0	sector8	0	-1.96	0.91	-2.17	0.0333	0.8734	-3.77	-0.16	-5.38	1.45
**β2-AAb*Sector**	sector2	0	sector7	0	3.82	1.82	2.09	0.0395	0.9044	0.19	7.45	-3.06	10.69
**β2-AAb*Sector**	sector2	0	sector8	1	4.01	1.93	2.08	0.0411	0.9108	0.17	7.85	-3.27	11.28
**β2-AAb*Sector**	sector2	0	sector9	1	3.90	1.94	2.01	0.0478	0.9333	0.04	7.76	-3.41	11.21
**β2-AAb*Sector**	sector2	1	sector5	1	0.84	0.42	2	0.0491	0.9367	0.00	1.67	-0.74	2.42
**β2-AAb*Sector**	sector3	1	sector8	1	2.41	0.39	6.18	<.0001	<.0001	1.63	3.18	0.94	3.88
**β2-AAb*Sector**	sector3	1	sector9	1	2.30	0.41	5.56	<.0001	<.0001	1.48	3.12	0.74	3.86
**β2-AAb*Sector**	sector4	1	sector5	1	1.66	0.25	6.66	<.0001	<.0001	1.17	2.16	0.72	2.60
**β2-AAb*Sector**	sector4	1	sector6	1	1.91	0.31	6.15	<.0001	<.0001	1.29	2.52	0.74	3.07
**β2-AAb*Sector**	sector4	1	sector7	1	1.85	0.33	5.56	<.0001	<.0001	1.19	2.52	0.60	3.11
**β2-AAb*Sector**	sector4	1	sector8	1	2.42	0.38	6.31	<.0001	<.0001	1.66	3.18	0.97	3.86
**β2-AAb*Sector**	sector4	1	sector9	1	2.31	0.41	5.63	<.0001	<.0001	1.49	3.13	0.76	3.86

** *interaction term significant p-value=0.037*

**Differences of Least Squares Means (Sector*ETA-AAb) for Peripapillary region****													
**Effect**	**Sector**	**ETA-AAb***	**_Sector**	**_ETA-AAb***	**Estimate**	**SE**	**t Value**	**Pr > |t|**	**Adj P**	**Lower**	**Upper**	**Adj Lower**	**Adj Upper**

**ETA-AAb**		0		1	-3.29	2.98	-1.1	0.2734	0.2734	-9.24	2.65	-9.24	2.65
**ETA-AAb*Sector**	sector1	1	sector4	1	-10.38	3.05	-3.4	0.0008	0.0177	-16.39	-4.37	-19.71	-1.05
**ETA-AAb*Sector**	sector1	0	sector4	0	-1.97	0.61	-3.21	0.0015	0.0321	-3.18	-0.76	-3.85	-0.09
**ETA-AAb*Sector**	sector1	0	sector4	1	-10.68	3.54	-3.02	0.0029	0.0565	-17.66	-3.70	-21.51	0.15
**ETA-AAb*Sector**	sector1	0	sector3	0	-1.74	0.61	-2.83	0.0051	0.093	-2.95	-0.53	-3.62	0.14
**ETA-AAb*Sector**	sector3	0	sector4	1	-8.94	3.54	-2.53	0.0122	0.1901	-15.92	-1.97	-19.78	1.89
**ETA-AAb*Sector**	sector4	0	sector4	1	-8.71	3.54	-2.46	0.0147	0.2186	-15.69	-1.73	-19.54	2.13
**ETA-AAb*Sector**	sector2	1	sector4	1	-7.23	3.05	-2.37	0.0185	0.2602	-13.24	-1.23	-16.56	2.10
**ETA-AAb*Sector**	sector2	0	sector4	1	-7.63	3.54	-2.16	0.0322	0.383	-14.61	-0.65	-18.47	3.20
**ETA-AAb*Sector**	sector2	0	sector3	0	1.31	0.61	2.13	0.0338	0.3958	0.10	2.52	-0.57	3.19
**ETA-AAb*Sector**	sector1	0	sector2	0	-3.05	0.61	-4.96	<.0001	<.0001	-4.26	-1.84	-4.93	-1.17

** *interaction term significant p-value=0.023*

Supplementary Table S3a, b:

All the significant GPCR-AAb values when the foveal avascular zone characteristics (FAZ) were introduced in the models. We presented the: (a) Type 3 Tests of Fixed Effects for the interactions; (b) differences of the least square means (LS means) with estimates, standard error (SE), upper and lower confidence interval (CI) and adjusted upper and lower CI for the main effects.

Table S3a:

	**Type 3 Tests of Fixed Effects**	**F Value**	**Pr > F**
SVP (covariate: FAZ of SVP)	FAZ of SVP*Noci-AAb	4.97	0.0287
SVP (covariate: FAZ of SVP)	FAZ of SVP*α1-AAb	22.53	<.0001
ICP (covariate: FAZ of SVP)	FAZ of SVP*Noci-AAb	9.12	0.0034
ICP (covariate: FAZ of SVP)	FAZ of SVP*β2-AAb	9.74	0.0025
ICP (covariate: FAZ of SVP)	FAZ of SVP*α1-AAb	14.93	0.0002
ICP (covariate: FAZ of SVP)	FAZ of SVP*M2-AAb	11.6	0.0011
DCP (covariate: FAZ of SVP)	FAZ of SVP*Noci-AAb	7.58	0.0074

DCP (covariate: FAZ of SVP)	FAZ of SVP*α1-AAb	8.74	0.0041
Peripapillary region (covariate: FAZ of SVP)	FAZ of SVP*Noci-AAb	5.45	0.0225
Peripapillary region (covariate: FAZ of SVP)	FAZ of SVP*β2-AAb	5.05	0.0277
Peripapillary region (covariate: FAZ of SVP)	FAZ of SVP*α1-AAb	10.75	0.0016
Peripapillary region (covariate: FAZ of SVP)	FAZ of SVP*M2-AAb	7.41	0.0082
SVP (covariate: FAZ of ICP)	FAZ of ICP*α1-AAb	31.84	<.0001
ICP (covariate: FAZ of ICP)	FAZ of ICP*β2-AAb	8.96	0.0037
ICP (covariate: FAZ of ICP)	FAZ of ICP*α1-AAb	24.82	<.0001
ICP (covariate: FAZ of ICP)	FAZ of ICP*M2-AAb	11.5	0.0011
DCP (covariate: FAZ of ICP)	FAZ of ICP*α1-AAb	12.45	0.0007
Peripapillary region (covariate: FAZ of ICP)	FAZ of ICP*Noci-AAb	8.32	0.0052
Peripapillary region (covariate: FAZ of ICP)	FAZ of ICP*β2-AAb	5.21	0.0256
Peripapillary region (covariate: FAZ of ICP)	FAZ of ICP*α1-AAb	21.13	<.0001
Peripapillary region (covariate: FAZ of ICP)	FAZ of ICP*M2-AAb	7.59	0.0075
SVP (covariate: FAZ of DCP)	FAZ of DCP*α1-AAb	84.7	<.0001
ICP (covariate: FAZ of DCP)	FAZ of DCP*β2-AAb	4.59	0.0354
ICP (covariate: FAZ of DCP)	FAZ of DCP*α1-AAb	67.75	<.0001
ICP (covariate: FAZ of DCP)	FAZ of DCP*M2-AAb	4.42	0.0389
DCP (covariate: FAZ of DCP)	FAZ of DCP*α1-AAb	55.49	<.0001
Peripapillary region (covariate: FAZ of DCP)	FAZ of DCP*α1-AAb	26.77	<.0001

Table S3b:

	Differences of Least Squares Means											
Covariates	Effect			Estimate	SE	t Value	Pr > |t|	Adj P	Lower	Upper	Adj Lower	Adj Upper
SVP (covariate: FAZ of SVP)	Noci-AAb	0	1	0.49	0.61	0.79	0.4306	0.4306	-0.74	1.71	-0.74	1.71
SVP (covariate: FAZ of SVP)	α1-AAb	0	1	2.53	0.69	3.70	0.0004	0.0004	1.17	3.90	1.17	3.90
ICP (covariate: FAZ of SVP)	Noci-AAb	0	1	0.15	0.57	0.26	0.7957	0.7957	-0.99	1.28	-0.99	1.28
ICP (covariate: FAZ of SVP)	β2-AAb	0	1	1.99	1.25	1.59	0.1152	0.1152	-0.50	4.47	-0.50	4.47
ICP (covariate: FAZ of SVP)	α1-AAb	0	1	2.02	0.66	3.08	0.0029	0.0029	0.71	3.33	0.71	3.33
ICP (covariate: FAZ of SVP)	M2-AAb	0	1	3.04	1.26	2.41	0.0182	0.0182	0.53	5.55	0.53	5.55
DCP (covariate: FAZ of SVP)	Noci-AAb	0	1	0.54	0.65	0.84	0.4061	0.4061	-0.75	1.83	-0.75	1.83
DCP (covariate: FAZ of SVP)	α1-AAb	0	1	2.23	0.79	2.83	0.006	0.006	0.66	3.80	0.66	3.80
Peripapillary region (covariate: FAZ of SVP)	Noci-AAb	0	1	-1.46	1.05	-1.39	0.1691	0.1691	-3.55	0.63	-3.55	0.63
Peripapillary region (covariate: FAZ of SVP)	β2-AAb	0	1	2.64	2.32	1.14	0.2595	0.2595	-1.99	7.27	-1.99	7.27
Peripapillary region (covariate: FAZ of SVP)	α1-AAb	0	1	3.40	1.19	2.85	0.0057	0.0057	1.02	5.78	1.02	5.78
Peripapillary region (covariate: FAZ of SVP)	M2-AAb	0	1	5.04	2.34	2.16	0.0345	0.0345	0.38	9.71	0.38	9.71
SVP (covariate: FAZ of ICP)	α1-AAb	0	1	2.35	0.66	3.54	0.0007	0.0007	1.03	3.68	1.03	3.68
ICP (covariate: FAZ of ICP)	β2-AAb	0	1	0.87	1.06	0.83	0.4118	0.4118	-1.23	2.97	-1.23	2.97
ICP (covariate: FAZ of ICP)	α1-AAb	0	1	1.87	0.61	3.05	0.0031	0.0031	0.65	3.10	0.65	3.10
ICP (covariate: FAZ of ICP)	M2-AAb	0	1	1.79	1.03	1.74	0.0851	0.0851	-0.25	3.84	-0.25	3.84
DCP (covariate: FAZ of ICP)	α1-AAb	0	1	2.07	0.75	2.77	0.007	0.007	0.58	3.55	0.58	3.55
Peripapillary region (covariate: FAZ of ICP)	Noci-AAb	0	1	-0.85	1.08	-0.78	0.4361	0.4361	-3.01	1.31	-3.01	1.31
Peripapillary region (covariate: FAZ of ICP)	β2-AAb	0	1	1.03	2.03	0.51	0.614	0.614	-3.03	5.09	-3.03	5.09
Peripapillary region	α1-AAb	0	1	3.05	1.15	2.65	0.0099	0.0099	0.75	5.34	0.75	5.34

(covariate: FAZ of ICP)												
Peripapillary region (covariate: FAZ of ICP)	M2-AAb	0	1	3.00	1.99	1.51	0.1352	0.1352	-0.96	6.96	-0.96	6.96
SVP (covariate: FAZ of DCP)	α1-AAb	0	1	9.22	0.93	9.93	<.0001	<.0001	7.37	11.07	7.37	11.07
ICP (covariate: FAZ of DCP)	β2-AAb	0	1	1.76	1.21	1.45	0.151	0.151	-0.66	4.18	-0.66	4.18
ICP (covariate: FAZ of DCP)	α1-AAb	0	1	7.67	0.88	8.75	<.0001	<.0001	5.92	9.41	5.92	9.41
ICP (covariate: FAZ of DCP)	M2-AAb	0	1	2.67	1.19	2.25	0.0275	0.0275	0.30	5.04	0.30	5.04
DCP (covariate: FAZ of DCP)	α1-AAb	0	1	8.47	1.07	7.89	<.0001	<.0001	6.34	10.61	6.34	10.61
Peripapillary region (covariate: FAZ of DCP)	α1-AAb	0	1	10.86	1.77	6.14	<.0001	<.0001	7.33	14.38	7.33	14.38
